# Lung microbiome community state types are associated with ventilator-associated pneumonia risk: a secondary analysis of the MicroNAV cohort

**DOI:** 10.1128/spectrum.01263-26

**Published:** 2026-10-08

**Authors:** Shiji Xiao, Qiufeng Zhuang, Guobin Cui, Yinglin Li

**Affiliations:** 1 Department of Paediatric Intensive Care Unit, The Affiliated Hospital of Putian University 632539, Putian, China; 2 Department of Paediatric Intensive Care Unit, Putian Children’s Hospital, Putian, China; 3 Department of General Medicine, The Affiliated Hospital of Putian University 632539, Putian, China; Nova Southeastern University, Fort Lauderdale, Florida, USA; State Key Laboratory for Animal Disease Control and Prevention, Lanzhou, Gansu, China

**Keywords:** ventilator-associated pneumonia, lung microbiome, community state types, bacterial burden, mechanical ventilation, ecological susceptibility

## Abstract

In the publicly available MicroNAV cohort, baseline diversity analyses did not distinguish patients who developed ventilator-associated pneumonia (VAP) from non-VAP patients, leaving it unresolved whether community-state structure contains risk information. In an independent secondary analysis, we reprocessed raw 16S rRNA gene sequencing data from bronchoalveolar lavage samples using DADA2 exact amplicon sequence variant inference and decontam quality control, and analyzed 75 at-risk mechanically ventilated patients with paired longitudinal sampling (40 VAP and 35 non-VAP). No baseline genus distinguished future VAP from non-VAP by DESeq2 or MaAsLin3 after adjustment for sex and Glasgow Coma Scale (all adjusted *P* > 0.05). Dirichlet multinomial mixture modeling identified three community state types (CSTs) with distinct ecological profiles. In cause-specific Cox regression, CST1, a high-burden, oral-associated state, was associated with higher VAP hazard than CST2, a low-burden, diverse state (hazard ratio, 2.85; 95% CI, 1.15–7.02, *P* = 0.023; Gray’s test, *P* = 0.042). Adding CST to a burden-only model improved fit (likelihood-ratio test, *P* = 0.041), whereas pathogen species cultured at VAP onset did not differ by baseline CST (simulated Fisher’s exact test, *P* = 0.98). During ventilation, 12 oral-associated genera declined while total bacterial burden remained stable (linear mixed model, *P* = 0.66), but the association between commensal loss and subsequent VAP was not consistent across abundance scales. Baseline IL-1β and TNF-α concentrations were explained primarily by bacterial burden rather than community composition. These findings suggest that baseline lower-airway ecological states capture VAP susceptibility information not resolved by single-taxon, diversity, or burden-only analyses.

**IMPORTANCE:**

In mechanically ventilated ICU patients without pulmonary infection at baseline, the early lower-airway ecosystem may contain information about subsequent ventilator-associated pneumonia (VAP) susceptibility. In this independent reanalysis of the public MicroNAV cohort, baseline lower-airway communities classified by Dirichlet multinomial mixture modeling resolved into ecological states with different subsequent VAP risk, while individual genera, bacterial burden alone, and the eventual cultured pathogen did not explain the signal. Mechanical ventilation produced shared community restructuring: oral-associated genera declined and other taxa expanded, yet total bacterial burden remained stable, indicating ecological replacement rather than bacterial clearance. This restructuring was not consistently VAP-specific, and apparent community state type-dependent differences in declining-genus loss partly reflected different starting proportions. Together, these findings extend the original cohort by separating starting state heterogeneity from ventilation-associated restructuring. They suggest that initially noninfected ventilated airways represent distinct host-microbial terrains that may differ in vulnerability to pneumonia.

## INTRODUCTION

Ventilator-associated pneumonia (VAP) remains a major complication of invasive mechanical ventilation (IMV), affecting 5%–40% of ventilated patients and contributing substantially to ICU morbidity and mortality (1, 2). Its pathogenesis involves micro-aspiration of oropharyngeal and gastric contents, impaired mucociliary clearance, endotracheal-tube biofilm formation, pathogen translocation, and altered host immune defense (3). These mechanisms imply that VAP susceptibility is unlikely to be explained simply by whether a potential pathogen is already present at intubation. The relevant signal may instead lie in the broader lower-airway ecosystem, where community composition, bacterial burden, and host inflammatory tone are jointly shaped.

This distinction is particularly important in the lower respiratory tract, a low-biomass ecosystem governed by immigration from the upper airways and elimination by host defenses, as described by the adapted island model (4, 5). In such samples, interpretation of relative abundance may benefit from consideration of total bacterial burden and community context. An apparently dominant pathogen-associated genus does not necessarily indicate true overgrowth; it may instead reflect reduced competing biomass or low overall bacterial burden. Conversely, communities with similar dominant taxa may differ markedly in burden, diversity, or inflammatory context. Prior work has shown that oral enrichment of the lower airways can shape pulmonary inflammatory tone, and that respiratory microbiome composition or bacterial burden carries clinical information in critically ill and mechanically ventilated patients (6–8). These observations provide a rationale for asking whether baseline bronchoalveolar lavage (BAL) ecological states stratify subsequent VAP risk beyond single taxa, diversity metrics, bacterial burden, or the pathogen eventually cultured at infection onset.

The MicroNAV study provided an important longitudinal data set linking lung microbiome composition, inflammatory markers, metabolism, and VAP development in mechanically ventilated ICU patients (9). The parent analysis found no significant baseline alpha- or beta-diversity differences between VAP and non-VAP patients, but showed that airway communities changed during ventilation and that inflammatory markers differed during follow-up. These findings prompted us to examine the baseline microbiome from a different perspective, asking whether lower-airway communities could be grouped into ecological states associated with subsequent VAP risk.

Here, we independently reanalyzed the public MicroNAV 16S rRNA gene sequencing data to address this question. We hypothesized that mechanically ventilated patients without pulmonary infection at baseline harbor distinct lower-airway community states with different subsequent VAP susceptibility. We characterized baseline airway community states, evaluated their relationship with subsequent VAP development, incorporated quantitative measures of bacterial burden, and examined whether ventilation-associated community restructuring and airway cytokine patterns accounted for the community state type (CST)-associated risk signal.

## RESULTS

### Baseline BAL microbiome composition does not distinguish patients who develop VAP

**Fig 1:**
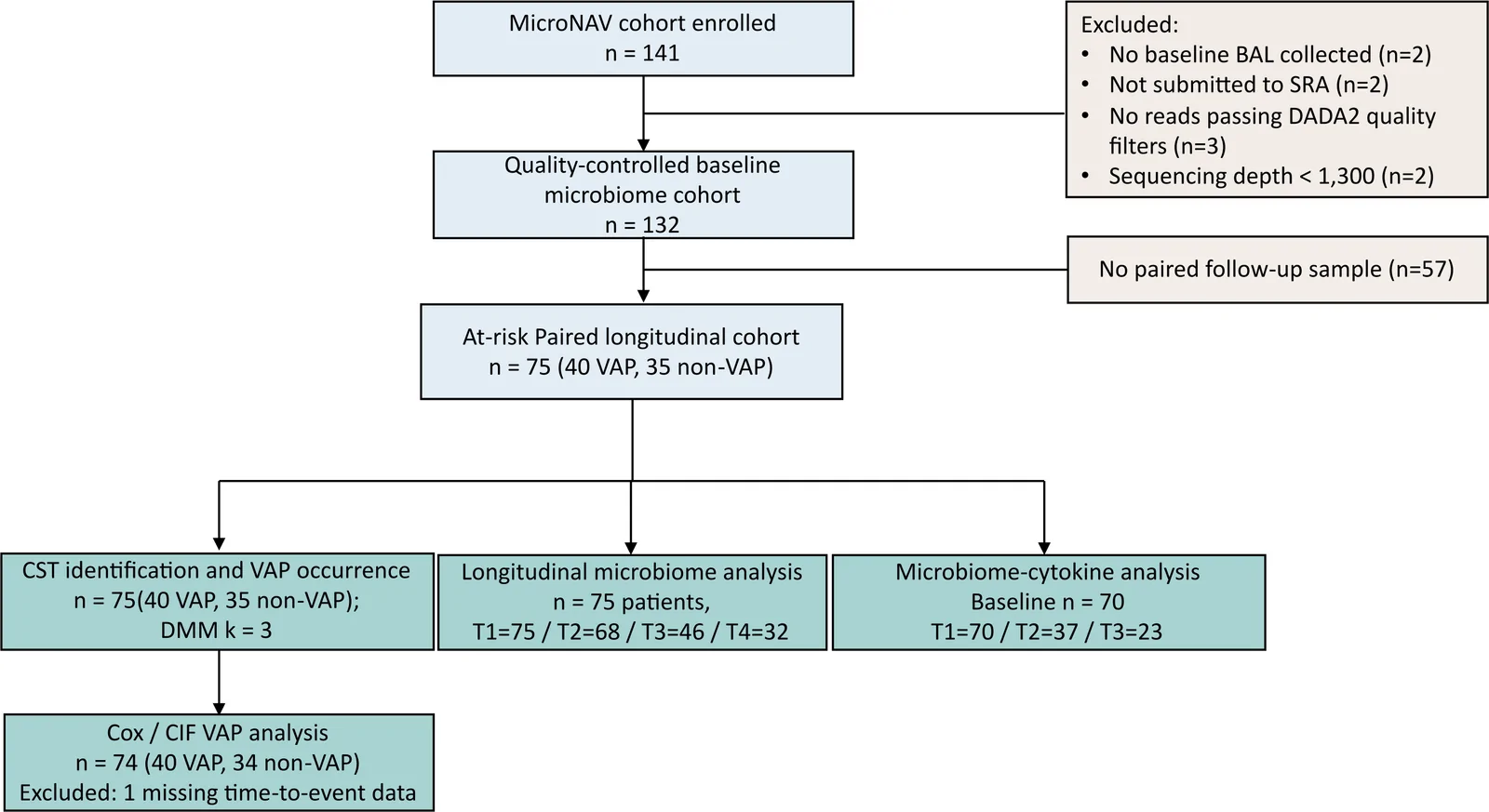
Patient flow and analytical cohort derivation.

Of 141 enrolled patients, 132 provided quality-controlled baseline BAL samples and 75 contributed paired longitudinal samples, constituting the at-risk cohort (40 VAP and 35 non-VAP; Fig. 1). Analysis-specific sample sizes are provided in Table S1. VAP patients were more frequently female (*P* = 0.048) and had lower Glasgow Coma Scale (GCS) scores at intubation (*P* = 0.04); both were included as covariates in all adjusted models. Baseline illness severity (Acute Physiology and Chronic Health Evaluation II [APACHE II], Sequential Organ Failure Assessment [SOFA]), Clinical Pulmonary Infection Score, and admission diagnoses were comparable between groups (Table S2).

At baseline, no genus-level feature distinguished patients who subsequently developed VAP from those who did not. DESeq2 identified no genera that were differentially abundant between patients who subsequently developed VAP and those who did not, either in the unadjusted model or in the model adjusted for sex and GCS (all adjusted *P* > 0.05; Fig. S1), with concordant results from MaAsLin3.

### Baseline communities resolved into three community state types with distinct ecological profiles

**Fig 2:**
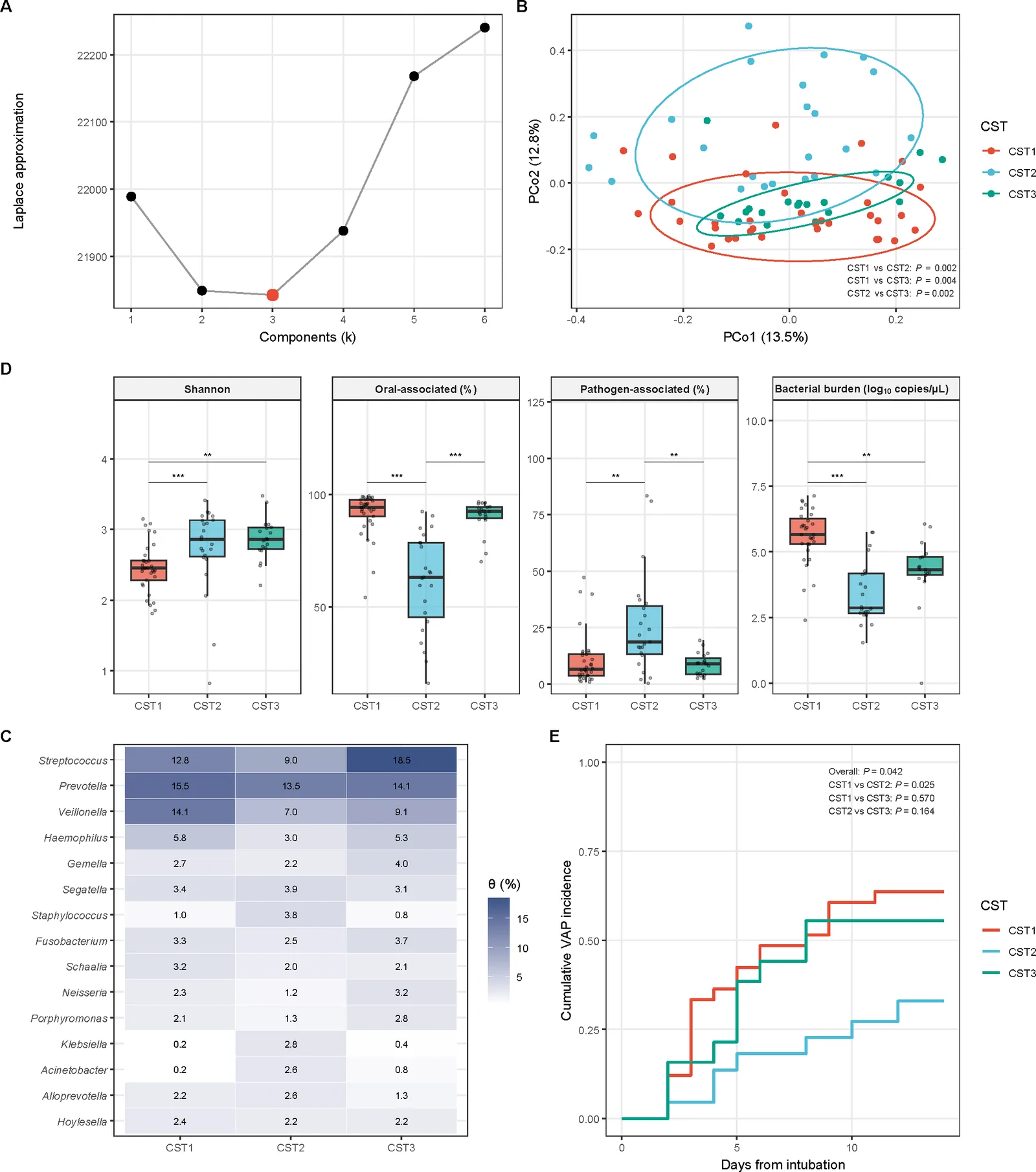
Baseline community state types stratify VAP risk. (**A**) Laplace approximation values for DMM models with *k* = 1–6 components; the minimum at *k* = 3 indicates the selected model. (**B**) Principal coordinates analysis of Bray–Curtis dissimilarity among baseline BAL communities, colored by CST assignment; ellipses indicate 80% data ellipses. (**C**) Heatmap of DMM-fitted genus weights (θ, %) for the top 15 genera across CSTs. (**D**) Baseline ecological profiles by CST, including Shannon diversity, oral-associated genera relative abundance, pathogen-associated genera relative abundance, and bacterial burden (16S, log_10_ copies/µL). Boxes show medians and interquartile ranges, with individual samples overlaid. Asterisks indicate Benjamini–Hochberg-adjusted pairwise *P* values: *, *P* < 0.05; **, *P* < 0.01; and ***, *P* < 0.001; exact values are provided in Table S4. (**E**) Cumulative incidence of VAP within the 14-day observation window by baseline CST, estimated with competing-risk methods. The overall Gray’s test and Benjamini–Hochberg-adjusted pairwise Gray’s *P* values are shown.

Dirichlet multinomial mixture (DMM) modeling of genus-level composition identified three CSTs (Laplace-optimal *k* = 3; all posterior probabilities >0.90; Fig. 2A). Bacterial burden, diversity, and inflammatory markers were not inputs to the clustering but were examined as properties of the resulting states. The three CSTs were comparable in baseline demographics, illness severity, and admission diagnosis (Table 1). Among in-ICU outcomes, only delirium incidence (*P* = 0.031) and IMV duration (*P* = 0.043) differed across groups, whereas ICU mortality, in-hospital mortality, and tracheostomy rates did not. In contrast, CSTs differed markedly across compositional, ecological, and inflammatory features. Baseline samples separated by CST in Bray–Curtis PCoA, while DMM-fitted genus weights revealed distinct community structures despite substantial overlap in dominant genera (Fig. 2B and C). Ecological profiles also differed significantly, with marked variation in bacterial burden and Shannon diversity across CSTs (Table 2; Fig. 2D), accompanied by corresponding differences in BAL cytokine concentrations (Fig. 4A); adjusted pairwise comparisons are provided in Table S4. Indicator species analysis further distinguished the states: CST1 and CST3 shared strong, high-abundance indicators, whereas CST2 was defined by weaker, lower-abundance indicators (Table 2). CST1 was a high-burden, oral-associated community with the highest inflammatory markers; CST3 was compositionally similar but had intermediate bacterial burden; and CST2 was a low-burden, more diverse community with the lowest inflammatory markers. Although CST2 had the highest relative abundance of the pathogen-associated genus set, it had the lowest ddPCR-anchored estimated absolute abundance of this set (Table S5).

**TABLE 1:** Baseline clinical characteristics and outcomes of the at-risk cohort stratified by baseline community state type^*c*^^,^^*d*^

Characteristic	Overall, *N* = 75^*a*^	CST1, *N* = 33^*a*^	CST2, *N* = 23^*a*^	CST3, *N* = 19^*a*^	*P* value^*b*^
Demographics					
Age, years	51 (34–70)	57 (34–71)	52 (30–70)	49 (38–67)	0.095
Sex					>0.999
Female	24 (32%)	11 (33%)	7 (30%)	6 (32%)	
Male	51 (68%)	22 (67%)	16 (70%)	13 (68%)	
BMI, kg/m²	25.5 (23.5–27.7)	26.6 (24.0–28.7)	24.6 (22.5–27.2)	25.6 (23.4–27.7)	0.303
Admission diagnoses					
Polytrauma	32 (43%)	17 (52%)	9 (39%)	6 (32%)	0.379
Post-operative	27 (36%)	11 (33%)	8 (35%)	8 (42%)	0.820
Intracranial hemorrhage	22 (29%)	9 (27%)	7 (30%)	6 (32%)	0.950
Hemorrhagic stroke	7 (9.3%)	2 (6.1%)	3 (13%)	2 (11%)	0.606
Ischemic stroke	6 (8.0%)	2 (6.1%)	2 (8.7%)	2 (11%)	0.867
Septic shock	7 (9.3%)	3 (9.1%)	2 (8.7%)	2 (11%)	>0.999
Hypovolemic shock	9 (12%)	5 (15%)	2 (8.7%)	2 (11%)	0.899
Comorbidities					
Arterial hypertension	26 (35%)	11 (33%)	9 (39%)	6 (32%)	0.862
Diabetes mellitus	7 (9.3%)	2 (6.1%)	3 (13%)	2 (11%)	0.614
Chronic kidney disease	6 (8.0%)	1 (3.0%)	3 (13%)	2 (11%)	0.364
Severity scores					
GCS	7 (6–10)	7 (6–8)	10 (6–14)	7 (6–9)	0.221
APACHE II	17 (11–20)	17 (14–20)	14 (9–20)	17 (12–21)	0.353
SOFA	8 (7–9)	8 (7–9)	8 (6–9)	8 (7–9)	0.939
CPIS	2 (1–4)	2 (1–4)	2 (1–4)	2 (1–3)	>0.999
Vital signs					
Heart rate, bpm	80 (66–98)	80 (66–96)	87 (78–104)	75 (51–96)	0.151
MAP, mmHg	102.7 (87.7–116.0)	96.7 (85.3–115.0)	109.3 (96.0–118.0)	103.3 (87.7–116.3)	0.692
Laboratory					
White blood cell count, ×10^3^/μL	12.9 (9.5–16.3)	14.6 (11.5–16.7)	11.8 (9.0–16.6)	11.8 (8.0–15.3)	0.152
Hemoglobin, g/dL	13.0 (11.4–14.0)	13.0 (11.6–14.0)	13.0 (11.4–14.3)	13.3 (11.7–14.5)	0.828
Platelets, ×10^3^/μL	200 (160–270)	200 (150–260)	180 (150–280)	250 (180–310)	0.175
Creatinine, mg/dL	1.0 (0.8–1.3)	0.9 (0.8–1.1)	1.2 (0.8–1.5)	0.9 (0.7–1.3)	0.473
Lactate, mmol/L	2.0 (1.2–3.2)	2.5 (1.4–4.9)	2.0 (1.0–3.2)	1.5 (0.9–2.6)	0.134
PaO_2_, mmHg	78 (70–94)	76 (72–88)	82 (68–95)	85 (69–95)	0.789
PaO_2_/FiO_2_ ratio	243 (200–294)	240 (173–293)	246 (214–305)	247 (217–309)	0.454
Treatment and outcomes					
Antibiotics within 24 h	29 (39%)	14 (42%)	10 (43%)	5 (26%)	0.464
Delirium during ICU	30 (40%)	12 (36%)	14 (61%)	4 (21%)	0.031
ICU mortality	17 (23%)	10 (30%)	3 (13%)	4 (21%)	0.320
In-hospital mortality	22 (29%)	13 (39%)	5 (22%)	4 (21%)	0.258
IMV duration, days	8 (5–11)	10 (6-16)	8 (5–10)	6 (4–10)	0.043
ICU LOS, days	13 (8–18)	15 (9–23)	13 (8–16)	11 (6–14)	0.237
VAP					0.101
Non-VAP	35 (47%)	12 (36%)	15 (65%)	8 (42%)	
VAP	40 (53%)	21 (64%)	8 (35%)	11 (58%)	

^*a*^ Data are median (Q1–Q3) or *n* (%).

^*b*^ *P* values were calculated using the Kruskal–Wallis rank-sum test for continuous variables and Fisher’s exact test for categorical variables.

^*c*^ APACHE II, Acute Physiology and Chronic Health Evaluation II; BMI, body mass index; CPIS, Clinical Pulmonary Infection Score; CST, community state type; GCS, Glasgow Coma Scale; IMV, invasive mechanical ventilation; LOS, length of stay; MAP, mean arterial pressure; SOFA, Sequential Organ Failure Assessment; VAP, ventilator-associated pneumonia.

^*d*^ Data missing: BMI, APACHE II, SOFA, CPIS, creatinine (*n* = 1 each); lactate (*n* = 4).

**TABLE 2:** Community state type ecological profile and indicator genera^*a*^

Property	CST1 (*n* = 33)	CST2 (*n* = 23)	CST3 (*n* = 19)	*P*
Ecological and inflammatory profile				
Shannon diversity	2.5 (2.4–2.6)	2.9 (2.6–3.2)	3.0 (2.8–3.1)	<0.001
Bacterial burden, copies/µL	4.5 × 10^5^ (1.9 × 10^5^ to 2.3 × 10^6^)	7.4 × 10^2^ (4.7 × 10^2^ to 1.5 × 10^4^)	2.1 × 10^4^ (1.3 × 10^4^ to 6.4 × 10^4^)	<0.001
IL-1β, pg/mL	1,876 (436–5,483)	4.3 (0.8–31)	1,359 (1.6–5,028)	<0.001
IL-6, pg/mL	17.8 (3.9–138.9)	2.4 (0.3–34.2)	26.4 (1.9–332.4)	0.022
TNF-α, pg/mL	114.4 (41–275)	3.5 (0.6–16)	35.0 (2.9–74)	<0.001
Indicator genera (IndVal.g, 999 permutations, all *P* < 0.05)				
*n* indicators	7	17	24	
IndVal.g range	0.75–0.95	0.55–0.95	0.55–0.95	
Indicator genera	*Neisseria*, *Pseudoleptotrichia*, *Lachnoanaerobaculum*, *Capnocytophaga*, *Bergeyella*, *Mogibacterium*, *Actinobacillus* (all shared with CST3; oral-associated)	*Lactobacillus* (0.72), *Lacticaseibacillus* (0.55); *Acinetobacter*, *Reyranella*, *Bradyrhizobium*, *Sediminibacterium*, *Klebsiella*, *Pseudomonas*, *Sphingomonas*, *Escherichia-Shigella*, *Methylobacterium*, *Fimbriiglobus*, *Tautonia*, *Peptoniphilus*, *Massilia*, *Proteus*, *Comamonas* (shared with CST3; environmental/nosocomial)	*Aggregatibacter* (0.83), *Oceanivirga* (0.55); *Neisseria*, *Pseudoleptotrichia*, *Lachnoanaerobaculum*, *Capnocytophaga*, *Bergeyella*, *Mogibacterium*, *Actinobacillus* (shared with CST1; oral-associated); *Acinetobacter*, *Reyranella*, *Bradyrhizobium*, *Sediminibacterium*, *Klebsiella*, *Pseudomonas*, *Sphingomonas*, *Escherichia-Shigella*, *Methylobacterium*, *Fimbriiglobus*, *Tautonia*, *Peptoniphilus*, *Massilia*, *Proteus*, *Comamonas* (shared with CST2; environmental/nosocomial)	

^*a*^ Continuous variables: median (IQR). Kruskal–Wallis test for continuous variables; cytokine *P* values BH-corrected. IndVal.g = generalized indicator value (0–1; higher = stronger association); 26 of 66 genera reached significance (999 permutations).

### Community state types stratify VAP risk

The VAP rate was 64% in CST1, 58% in CST3, and 35% in CST2. Within the 14-day window (74 of 75 patients evaluable; Table S1), 38 had a VAP event, 6 died on ventilation, 26 were extubated, and 4 were censored. In cause-specific Cox regression, with CST2 as the reference, CST1 was associated with a higher VAP hazard (hazard ratio [HR], 2.85; 95% CI, 1.15–7.02; *P* = 0.023), whereas CST3 showed a nonsignificant elevation (HR, 2.35; 95% CI, 0.86–6.40; *P* = 0.095; Table 3). The cumulative incidence of VAP separated the states concordantly (Gray’s test, *P* = 0.042; Fig. 2E). The association remained similar across sensitivity models, additionally accounting for age, SOFA, PaO_2_/FiO_2_, bacterial burden, and antibiotic administration within the first 24 h (Table 3). To test whether the CST–VAP association was independent of bacterial quantity, we added bacterial-burden tertiles to the CST-based Cox model. CST1 remained associated with VAP risk (HR, 4.29; *P* = 0.014), whereas bacterial burden showed no graded association with VAP (mid vs low tertile: HR, 0.66; *P* = 0.433; high vs low tertile: HR, 0.97; *P* = 0.952). Adding CST to a burden-only model improved model fit (likelihood-ratio test, *P* = 0.041; Table S6), supporting that baseline community structure stratified VAP risk beyond bacterial quantity alone.

**TABLE 3:** Cox proportional hazard models for time to VAP by baseline community state type^*a*^

Model	CST1 (*n* = 33)		CST3 (*n* = 19)	
	HR (95% CI)	*P*	HR (95% CI)	*P*
Unadjusted	3.27 (1.36–7.86)	0.008	2.74 (1.03–7.27)	0.043
Sex, GCS	2.85 (1.15–7.02)	0.023	2.35 (0.86–6.40)	0.095
Sex, GCS, age, SOFA	2.56 (1.03–6.38)	0.044	2.20 (0.80–6.04)	0.126
Sex, GCS, PaO_2_/FiO_2_	2.77 (1.11–6.88)	0.028	2.32 (0.85–6.31)	0.101
Sex, GCS, bacterial burden	3.80 (1.15–12.52)	0.028	2.46 (0.79–7.65)	0.120
Sex, GCS, antibiotics within 24 h	2.79 (1.10–7.03)	0.030	2.16 (0.77–6.04)	0.141
Sex, GCS, age, SOFA, PaO_2_/FiO_2_, bacterial burden, antibiotics within 24 h	4.22 (1.24–14.39)	0.022	2.47 (0.78–7.84)	0.125

^*a*^ CST2 serves as the reference category, sequentially adding covariates. HR, hazard ratio; CI, confidence interval; GCS, Glasgow Coma Scale; SOFA, Sequential Organ Failure Assessment.

### VAP risk tracked community state rather than a single taxonomic feature

CST1 and CST3 were compositionally closer to each other than to CST2 (Bray–Curtis dissimilarity 0.18 vs 0.34–0.35) and had higher VAP rates, whereas CST2 had the lowest rate despite higher relative abundance of genera commonly associated with nosocomial infection (Table 2). Baseline 16S showed some correspondence with the genus subsequently cultured at VAP onset, but this correspondence was not related to baseline CST (Table S8). The pathogen cultured at VAP onset likewise did not differ by baseline CST, either by Gram category (Fisher’s exact test, *P* = 0.17) or by species identity (simulated Fisher’s exact test, *P* = 0.98; Table S7).

### Mechanical ventilation reshapes baseline community structure while total bacterial burden is conserved

#### The baseline stratification does not persist under ventilation

At baseline, the three CSTs were compositionally distinct (PERMANOVA, *R*² = 0.11; *P* = 0.001). By the first follow-up, baseline CST labels no longer structured the community (*R*² = 0.036; *P* = 0.10), indicating that the baseline stratification dissolved under ventilation rather than persisting.

#### Community composition shifts in both VAP and non-VAP patients while total burden is conserved

**Fig 3:**
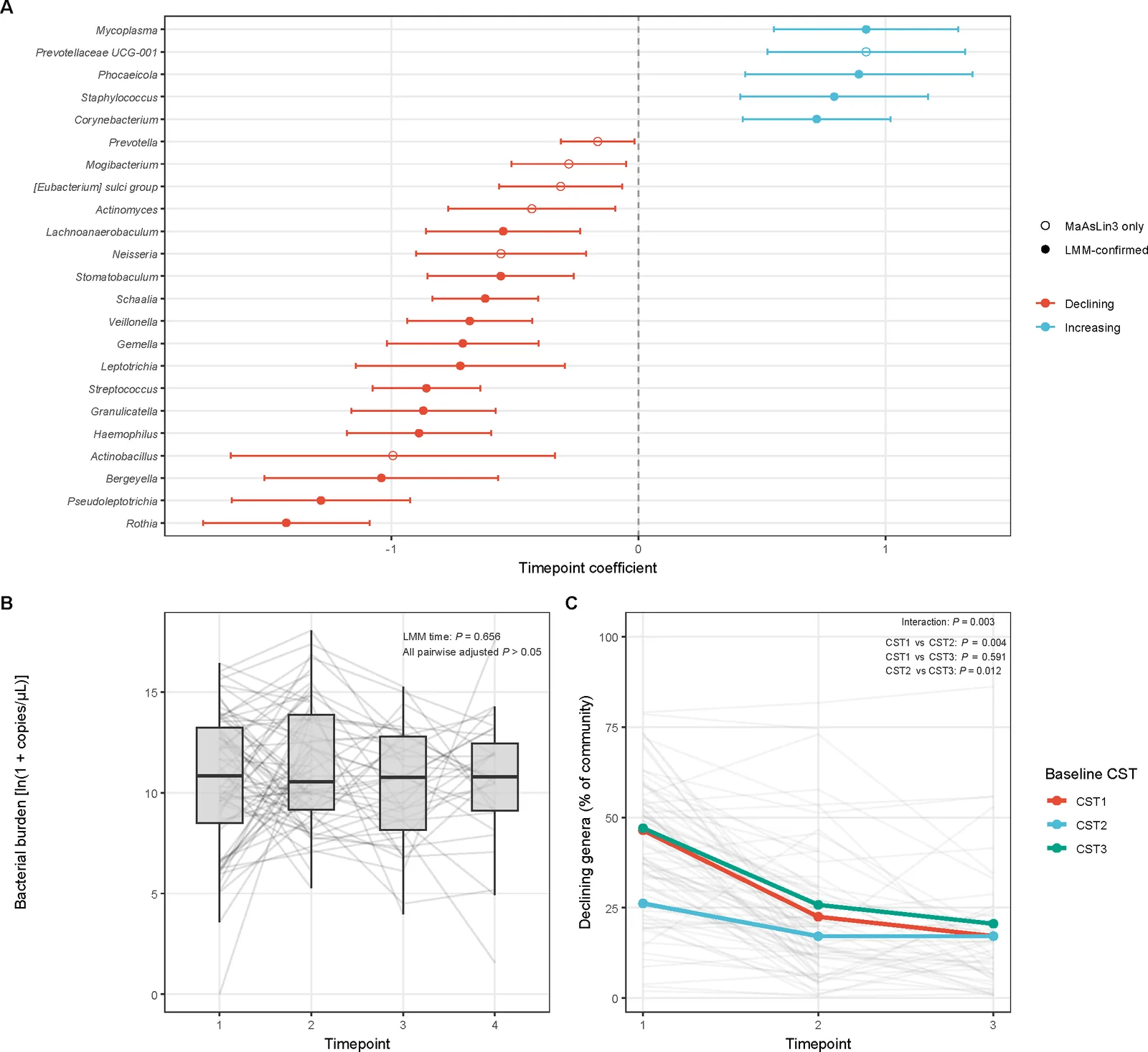
Mechanical ventilation reshapes airway community composition while total bacterial burden is conserved. (**A**) MaAsLin3 timepoint coefficients for genera changing during ventilation. Negative and positive coefficients indicate decreasing and increasing genera, respectively; bars show 95% CIs. Filled points indicate genera also confirmed by linear mixed models. (**B**) Longitudinal total bacterial burden, shown as ln(1 + copies/µL). Gray lines connect paired samples. (**C**) Longitudinal change in the declining-genus proportion by baseline CST. Gray lines show individual trajectories, and colored lines show CST-specific means.

Under mechanical ventilation, 12 oral-associated genera, spanning obligate anaerobes, facultative anaerobes, and aerobes, showed concordant declines in MaAsLin3 and linear mixed models (Table 4; Fig. 3A). Four genera increased in relative abundance; *Mycoplasma*, *Staphylococcus*, and *Phocaeicola* also increased in estimated absolute abundance, whereas *Corynebacterium* did not. Total bacterial burden did not change over time (linear mixed model [LMM], *P* = 0.66; Fig. 3B), indicating that these shifts represent compositional restructuring rather than net biomass loss. All 12 declining genera also decreased on the estimated absolute-abundance scale (all *P* < 0.001; Table 4).

**TABLE 4:** Genera with temporal change during mechanical ventilation and corresponding estimated absolute-abundance analyses

Genus	MaAsLin3		LMM		Estimated absolute abundance		eHOMD oral-associated	O_2_ tolerance
	*β*	*P* ^a^	*β*	*P* ^*a*^	*β*	*P* ^*a*^		
Decreasing (*n* = 12)								
*Rothia*	−1.43	<0.001	−0.0036	<0.001	−1.69	<0.001	Yes	Aerobe
*Granulicatella*	−0.87	<0.001	−0.0030	<0.001	−1.44	<0.001	Yes	Facultative
*Pseudoleptotrichia*	−1.29	<0.001	−0.0034	<0.001	−1.26	<0.001	Yes	Obligate anaerobe
*Haemophilus*	−0.89	<0.001	−0.0117	0.027	−1.19	<0.001	Yes	Facultative
*Gemella*	−0.71	<0.001	−0.0052	0.003	−1.14	<0.001	Yes	Facultative
*Leptotrichia*	−0.72	0.005	−0.0013	0.002	−1.12	<0.001	Yes	Obligate anaerobe
*Lachnoanaerobaculum*	−0.55	0.002	−0.0007	<0.001	−1.06	<0.001	Yes	Obligate anaerobe
*Bergeyella*	−1.04	<0.001	−0.0010	0.013	−1.01	<0.001	Yes	Aerobe
*Streptococcus*	−0.86	<0.001	−0.0309	<0.001	−0.96	<0.001	Yes	Facultative
*Veillonella*	−0.68	<0.001	−0.0247	<0.001	−0.84	<0.001	Yes	Obligate anaerobe
*Schaalia*	−0.62	<0.001	−0.0043	<0.001	−0.81	<0.001	Yes	Facultative
*Stomatobaculum*	−0.56	0.001	−0.0010	0.005	−0.66	<0.001	Yes	Obligate anaerobe
Increasing (*n* = 4)								
*Mycoplasma*	0.92	0.003	0.0089	0.022	0.83	<0.001	No	Variable
*Staphylococcus*	0.79	0.023	0.0192	0.013	0.43	0.044	No	Facultative
*Phocaeicola*	0.89	0.038	0.0008	0.003	0.41	0.014	No	Obligate anaerobe
*Corynebacterium*	0.72	0.010	0.0040	0.005	0.35	0.088	No	Facultative

^*a*^ FDR adjusted across genera; values are *β* coefficients with corresponding *P* values, absolute-abundance *P* values are nominal. Estimated absolute abundance was calculated by multiplying relative abundance by the sample-matched ddPCR-derived total bacterial burden. Oral association was defined using eHOMD body-site annotations. Oxygen tolerance was assigned at the genus level from taxonomic descriptions and should be considered putative. CLR, centered log-ratio; ddPCR, droplet digital PCR; LMM, linear mixed model.

The association between this restructuring and subsequent VAP was not consistent across abundance scales (Table S9), and no genus showed a significant timepoint × VAP interaction after FDR correction. The baseline declining-genus score also was not associated with VAP risk when modeled as a continuous predictor (HR, 1.13; *P* = 0.52), and adding the score to the CST-based model did not suggest that this score explained the CST-associated risk signal (HR, 0.63, *P* = 0.09). In contrast, the magnitude of declining-genus loss varied by baseline community configuration. Communities that entered ventilation with a higher proportion of declining genera showed larger declines (baseline CST × timepoint interaction, *P* = 0.003; Fig. 3C), whereas neither the proportion of increasing genera (*P* = 0.148) nor Shannon diversity (*P* = 0.449) showed a CST-dependent trajectory. This pattern was reflected across several individual declining genera (7 of 12 genera with interaction FDR < 0.05; Table S10).

#### Associations between airway inflammation and the microbiome

**Fig 4:**
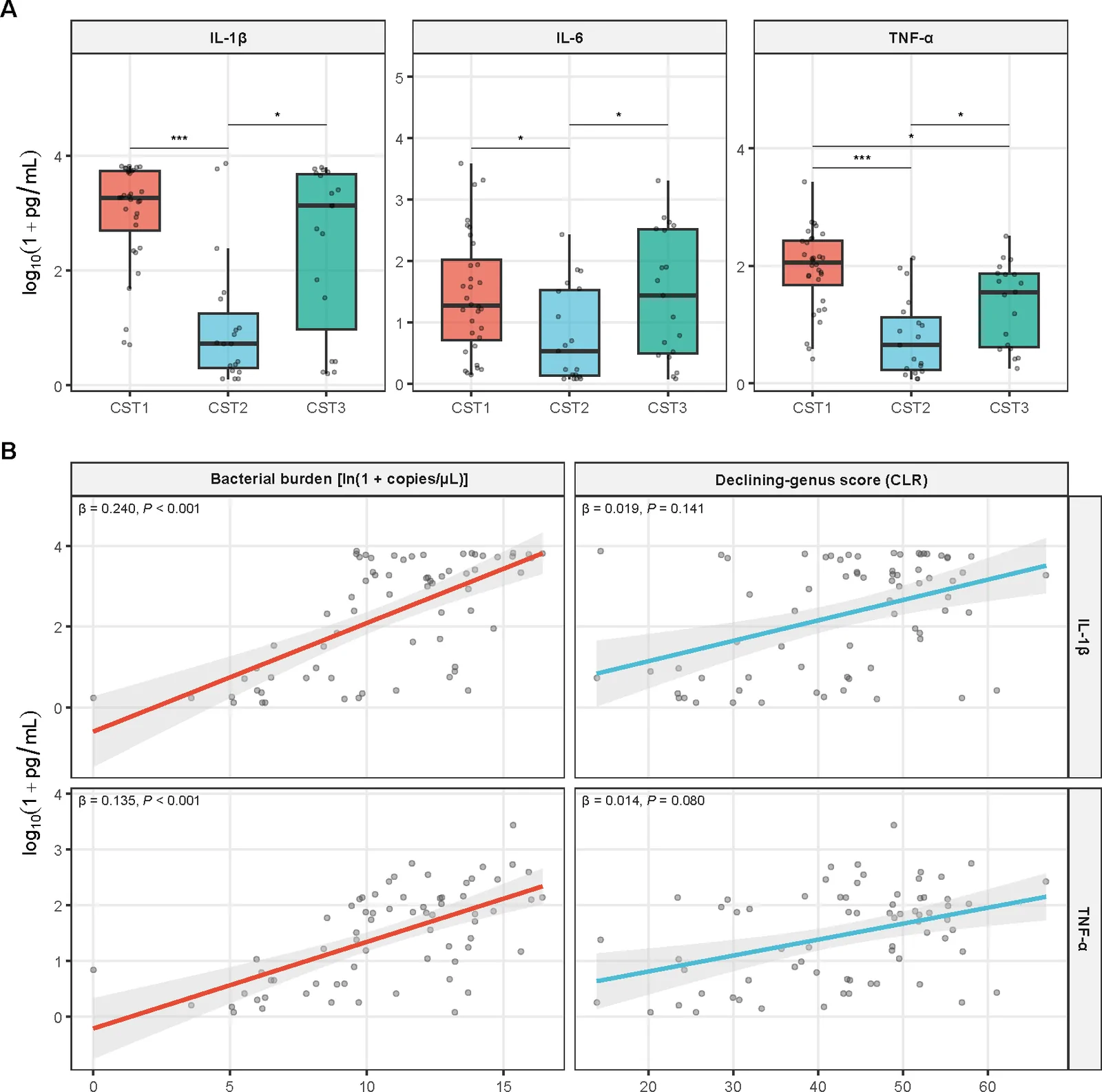
Baseline airway inflammation is associated primarily with bacterial burden. (**A**) BAL IL-1β, IL-6, and TNF-α concentrations by baseline CST, shown as log_10_(1 + pg/mL). Boxes show medians and interquartile ranges; points show individual samples. Asterisks indicate Benjamini–Hochberg-adjusted pairwise *P* values: *, *P* < 0.05; **, *P* < 0.01; and ***, *P* < 0.001; exact values are provided in Table S4. (**B**) Baseline associations of IL-1β and TNF-α with bacterial burden and the declining-genus score. Lines show linear fits with 95% confidence bands.

BAL cytokine concentrations were highest in CST1 and lowest in CST2 (IL-1β and TNF-α, *P* < 0.001; IL-6, *P* = 0.022; Fig. 4A). To determine whether these differences were driven primarily by bacterial quantity or community composition, we regressed each baseline cytokine on bacterial burden, the declining-genus score, sex, and GCS (Table S11). These models explained a substantial share of IL-1β and TNF-α variance (*R*² = 0.47 and 0.43) but little IL-6 variance (*R*² = 0.14). For IL-1β and TNF-α, bacterial burden was the dominant term (both *P* < 0.001; Fig. 4B), whereas the declining-genus score was not independently associated with either cytokine after adjustment for burden (IL-1β, *P* = 0.14; TNF-α, *P* = 0.08). In contrast, the IL-6 model showed a weak association with the declining-genus score (*P* = 0.03) rather than bacterial burden (*P* = 0.32); given the low explained variance and nominal, uncorrected significance, this isolated IL-6 signal should be interpreted cautiously. Thus, baseline IL-1β and TNF-α, the cytokines most elevated in the high-risk CST1, were more closely associated with bacterial quantity than with community composition.

In longitudinal models with a declining-genus score × timepoint interaction, the interaction coefficients were negative for all three cytokines, and none reached significance (IL-1β, *P* = 0.22; IL-6, *P* = 0.07; TNF-α, *P* = 0.19); these models had limited power given the sample sizes at later timepoints (*n* = 70, 37, 23).

### Sensitivity analyses

Re-clustering at *k* = 2 and 4 preserved both a low-risk and a high-risk state with concordant assignment (Table S12), and re-deriving CSTs in the full 132-patient baseline cohort and evaluating the resulting assignments in the at-risk cohort preserved the ordering of VAP risk with attenuated hazard ratios (Table S12). Adjusting longitudinal models for early antibiotic exposure did not alter the set of dual-confirmed declining genera. A logistic regression was concordant (CST1 OR, 3.28, *P* = 0.036 unadjusted; OR, 2.46, *P* = 0.15 adjusted for sex and GCS).

## DISCUSSION

Building on the longitudinal MicroNAV cohort of mechanically ventilated ICU patients, this secondary analysis shows that the baseline lower-airway microbiome contains ecological information relevant to subsequent VAP susceptibility that was not apparent from single-genus or diversity-based comparisons. Rather than identifying a uniform pathogen signature, our analysis revealed heterogeneity in baseline BAL community structure, bacterial burden, and inflammatory tone. The baseline signal therefore appears to precede clinical infection and to reside in the organization of the lower-airway community rather than in any single organism.

Baseline BAL communities separated into three CSTs with distinct ecological profiles and different subsequent VAP rates. These states were not defined by bacterial burden, diversity, or cytokine concentrations, because these variables were not used for DMM clustering; nevertheless, they differed markedly after CST assignment. CST1 was characterized by high bacterial burden, low diversity, a high proportion of oral-associated genera, and the highest VAP rate. CST2, by contrast, had the lowest bacterial burden, greater diversity, the weakest inflammatory profile, and the lowest VAP rate, despite a higher relative abundance of genera commonly associated with nosocomial infection. This apparent paradox illustrates the limitations of interpreting relative-abundance data alone in low-biomass respiratory samples, consistent with prior concerns about relative abundance and biomass effects in microbiome studies (10). In such samples, a high relative abundance of pathogen-associated taxa may reflect low competing biomass rather than high absolute pathogen load. Quantitative calibration by ddPCR was therefore important for interpreting the biological meaning of community structure, in line with quantitative microbiome approaches (11).

These results argue against a simple model in which VAP susceptibility is determined by the baseline abundance of a single pathogen or pathogen-associated genus. Genus-level analyses did not identify taxa that distinguished patients who later developed VAP from those who did not, and the organism cultured at VAP onset did not differ across baseline CSTs. This disconnect between baseline ecological state and eventual pathogen identity suggests that CSTs should not be interpreted as direct surrogates for the infecting organism. Instead, they may represent ecological phenotypes of the lower airway: integrated states shaped by microbial immigration, elimination, local growth conditions, and host defense (5, 12). Within the adapted island model of the respiratory tract, a high-burden, oral-associated state such as CST1 is consistent with a baseline environment in which microbial immigration or persistence outweighs elimination (5). Such a state may reflect an airway terrain shaped by aspiration tendency, impaired mucociliary or immune clearance, epithelial injury, and other host or mechanical factors, all recognized contributors to VAP susceptibility (6, 13). Because baseline BAL was collected within 12 h of intubation, CST1 may also partly reflect airway processes accompanying the acute illness and intubation rather than a wholly pre-existing susceptibility state. Its association with VAP nevertheless persisted after adjustment for measured neurologic and physiologic severity.

Mechanical ventilation then appeared to impose a common ecological disturbance on this baseline landscape. The baseline CST structure was no longer evident by the first follow-up, and ventilation was associated with oral-associated genus depletion, expansion of non-oral genera, and conserved total bacterial burden. This pattern is most consistent with ecological replacement rather than net bacterial clearance. The restructuring occurred in both VAP and non-VAP patients, and its association with VAP was not consistent across abundance scales; no genus showed a significant VAP-specific temporal interaction after FDR correction. The declining-genus score also did not predict VAP. Prior studies have similarly described respiratory microbiome restructuring during intubation or mechanical ventilation (14, 15). Thus, the ventilation-associated trajectory is better interpreted as a shared response to mechanical ventilation than as the source of the VAP-risk signal. The larger percentage-point declines observed in CST1 and CST3 likely reflect different starting proportions rather than an independent mechanism of VAP susceptibility; per-genus analyses were concordant, although communities richer in these taxa at baseline inherently have greater scope for decline. In this sense, mechanical ventilation may erase the initial community-state structure, whereas the risk-relevant information resides in the baseline ecological configuration.

Cytokine modeling further separated bacterial quantity from the CST-associated VAP risk signal. Baseline IL-1β and TNF-α concentrations were explained primarily by bacterial burden, whereas the declining-genus score was not independently associated with them after adjustment for burden. This pattern is compatible with innate immune sensing of microbial load and with prior evidence that oral enrichment of the lower airways can shape mucosal inflammatory tone (3, 7). However, we cannot exclude that shared upstream factors such as mucosal barrier integrity, aspiration burden, or ventilator settings jointly influence both microbial composition and local cytokine concentrations. Bacterial burden did not account for the CST–VAP association. This was most apparent in CST2, which had the lowest burden and weakest inflammatory profile, yet its lower VAP rate could not be attributed simply to bacterial quantity. Thus, burden explained much of the inflammatory signal while leaving the CST-associated difference in VAP risk still unresolved. Baseline cytokines may primarily reflect the quantitative microbial burden present at sampling, whereas CSTs capture broader ecological organization that may include colonization resistance, niche occupancy, and host–microbe interactions not fully represented by the measured cytokines, a framing consistent with ecological and host–microbe models of the lung microbiome (6, 7, 12).

Our findings extend the parent MicroNAV study, which reported longitudinal microbiome shifts during mechanical ventilation and elevated follow-up cytokines in VAP patients but did not identify baseline diversity or genus-level differences between VAP and non-VAP patients (9). The present analysis suggests that the absence of a single-taxon or diversity signal does not imply absence of microbiome-derived risk information. Rather, the signal became apparent only when baseline communities were considered as ecological states and interpreted alongside bacterial burden. This interpretation is broadly aligned with prior work showing that airway community states, bacterial burden, and respiratory microbiome profiles can carry clinical or inflammatory information in critically ill and mechanically ventilated patients (14–17). At the same time, the specific taxa associated with higher-risk states appear to vary across cohorts. In this cohort, the higher-risk state was high-burden and oral-associated rather than simply enriched in pathogen-associated genera. Together with prior studies emphasizing VAP heterogeneity and variable respiratory microbiome signatures (13, 16, 17), these findings suggest that the recurrent theme may not be a universal VAP-associated taxonomic signature, but the existence of heterogeneous host–microbial airway terrains that shape vulnerability during mechanical ventilation.

This study should be interpreted within the broader context of the multifactorial pathogenesis of VAP. Microbial community structure, bacterial burden, aspiration, airway clearance, immune competence, antibiotic exposure, and device-associated ecological disruption likely interact dynamically during mechanical ventilation (13, 15). Our results do not establish that baseline CSTs cause VAP, nor do they imply that CSTs can directly predict the infecting pathogen. Rather, they support the hypothesis that the baseline airway ecosystem contains information about susceptibility that is lost when the microbiome is reduced to individual taxa or relative abundance alone.

Several limitations should be acknowledged. This was a secondary analysis of a single-center cohort composed mainly of neurological and trauma ICU patients, so generalizability to broader ICU populations is uncertain. The observational design precludes causal inference. Aspiration and airway-protection status were not directly measured. Antibiotic information was limited to a binary indicator of whether antibiotics were administered in the first 24 h; antibiotic class, indication, exact timing relative to intubation, duration, subsequent treatment changes, and culture-directed therapy were unavailable. The contribution of differential antibiotic management to the observed CST–VAP associations therefore cannot be fully assessed. Residual confounding by immunosuppression, airway clearance, and other factors affecting both the airway microbiome and VAP risk also cannot be excluded. Event times in the competing-risks models were recorded with day-level precision, adding imprecision to time-to-event estimates. 16S rRNA V4 sequencing provides genus-level resolution and cannot assess strain-level virulence, antimicrobial resistance, or functional pathways (10). BAL sampling captures the distal airway but may miss regional heterogeneity within the lung. DMM modeling imposes discrete states on what may be a continuum of ecological configurations, and estimates for rare genera may remain unstable despite prevalence filtering and multiple random initializations (18). Finally, cytokine analyses were limited by sample availability at later timepoints, reducing power to characterize longitudinal microbiome–inflammation relationships.

In conclusion, baseline BAL communities in mechanically ventilated patients resolved into ecological states with different subsequent VAP risk. The signal was not captured by individual genera, pathogen-associated relative abundance, bacterial burden alone, or the microbial trajectory after ventilation. These results do not define a fixed microbial signature of VAP; rather, they suggest that VAP susceptibility may arise from heterogeneous host–microbial airway terrains that are best interpreted using both community structure and bacterial burden. Replication in larger, multi-center cohorts will be needed to determine whether these states represent recurrent ecological phenotypes or cohort-specific manifestations of a broader airway-susceptibility state.

## MATERIALS AND METHODS

### Study design and data source

This is a secondary analysis of publicly available data from the MicroNAV prospective observational cohort (BioProject PRJNA1212098; PhysioNet v1.1.1) (9, 19). The parent study enrolled 141 adult ICU patients (≥18 years) requiring mechanical ventilation for acute non-infectious respiratory failure at Clínica Universidad de la Sabana, Chía, Colombia (January 2020 to July 2022). BAL samples were collected within 12 h of intubation at baseline and at protocol-defined follow-up timepoints, including sampling at VAP diagnosis when applicable. VAP was diagnosed according to IDSA/ATS guidelines. Exclusion criteria included pregnancy, breastfeeding, prior IMV > 24 h, recent pre-enrollment antibiotic exposure, clinical or radiological evidence of pulmonary infection at baseline, chronic lung disease, and COVID-19-related respiratory failure. The reanalysis was conducted independently; the original investigators had no role in the design, analysis, or interpretation of the present study.

### 16S rRNA gene sequencing and decontamination

Raw paired-end reads (2 × 250 bp, Illumina MiSeq, V4 region) were reprocessed using DADA2 v1.38 (20) for exact amplicon sequence variant (ASV) inference [truncLen = c(220, 200), maxN = 0, maxEE = c(2, 5), truncQ = 2], yielding 13,733 ASVs across 1,044 samples (including 38 extraction–isolation negative controls) with 21,405,543 total quality-filtered reads (median, 18,004 per sample). Taxonomy was assigned against the SILVA v138.2 database and aggregated to genus level. Contamination was assessed using the decontam package (21) with the combined frequency–prevalence method (threshold = 0.1), incorporating ddPCR-derived bacterial 16S rRNA gene copy concentrations and 38 extraction–isolation negative controls. Forty-six ASVs were identified as contaminants and removed (Table S13). After removal of mitochondrial/chloroplast and zero-count ASVs, 12,843 ASVs were retained across 839 experimental samples with ≥1,300 quality-filtered reads (287 BAL, 289 tracheal, 263 nasal; Table S14).

### Statistical analysis

#### Baseline comparisons

Between-group comparisons of baseline characteristics used Wilcoxon rank-sum tests for continuous variables and Fisher’s exact tests for categorical variables. Genus-level differential abundance between VAP and non-VAP groups at baseline was assessed using DESeq2 (22) with poscounts size-factor estimation and normal shrinkage, and MaAsLin3 (23) with TSS normalization and LOG transformation; genera with prevalence < 10% were excluded. Two models were fitted: unadjusted (Model A) and adjusted for sex and GCS (Model B), the two variables that differed between groups at baseline.

#### Community state typing

DMM models (18) were fitted to the baseline genus-level count matrix of the at-risk cohort (*n* = 75) for *k* = 1–6 components, after excluding genera with prevalence < 10% or mean relative abundance < 0.1% (66 genera retained). Bacterial burden, diversity, and cytokines were not used in the clustering. For each *k*, the model was fitted from 30 random initializations, and the fit with the lowest Laplace approximation was retained. Each sample was assigned to the CST with the highest posterior probability; all exceeded 0.90. CSTs were characterized *post hoc* using normalized Dirichlet weights (θ%), indicator species analysis (IndVal.g, indicspecies package) (24), and sample-level ecological profiles including the proportion of oral-associated genera and pathogen-associated genera (Table S3). Oral-associated genera were defined from eHOMD body-site annotations (25, 26), whereas the pathogen-associated genera were prespecified based on the IDSA/ATS framework and supporting respiratory microbiology literature (27). The two sets were not mutually exclusive; definitions and genus membership are provided in Table S3. Bacterial burden and Shannon diversity were compared across CSTs by Kruskal–Wallis tests. Pairwise compositional dissimilarity between CSTs was quantified as the Bray–Curtis dissimilarity between CST centroids. Compositional separation of CSTs at baseline and first follow-up was tested by PERMANOVA on Bray–Curtis dissimilarities (999 permutations). Pairwise CST comparisons used PERMANOVA for Bray–Curtis composition and Dunn’s tests for continuous ecological measures, with Benjamini–Hochberg adjustment within each outcome.

#### Longitudinal differential abundance

Temporal genus-level changes were assessed using MaAsLin3 (primary; patient as a random effect, timepoint as integer-coded fixed effect, with VAP status included as a covariate) and confirmatory LMMs fitted with lme4/lmerTest using Satterthwaite degrees of freedom and Benjamini–Hochberg FDR correction. The LMM included a timepoint × VAP interaction term to test whether temporal trajectories differed by group. Genera significant in both methods (MaAsLin3, *q* < 0.05 and LMM, FDR < 0.05) constituted the dual-confirmed declining and increasing sets. A declining-genus score was computed as the sum of centered log-ratio (CLR)–transformed relative abundances of the dual-confirmed declining genera. Prior to CLR transformation, count zeros were replaced using Bayesian multiplicative replacement (geometric Bayesian multiplicative method; zCompositions::cmultRepl), after restricting each composition to genera present at ≥10% prevalence. Estimated absolute abundances were calculated by multiplying relative abundances by ddPCR-derived 16S rRNA gene copy concentrations. Total bacterial burden was tested for temporal change by LMM. To assess whether baseline community configuration influenced temporal trajectories, baseline-CST × timepoint interactions were tested on the declining-genus proportion, increasing-genus proportion, and Shannon diversity, and on each dual-confirmed declining genus individually (Benjamini–Hochberg-corrected).

#### CST–VAP association

Cause-specific Cox regression was used with VAP as the event of interest within a 14-day observation window; extubation and death on ventilation were treated as competing events. The CST with the lowest median bacterial burden was pre-specified as the reference. The primary model adjusted for sex and GCS; sensitivity models additionally adjusted for age, SOFA, PaO_2_/FiO_2_ ratio, antibiotic administration within the first 24 h, and log-transformed bacterial burden, each entered individually and in a combined model. Cumulative incidence was estimated by the Aalen–Johansen method and compared across CSTs by Gray’s test, with Benjamini–Hochberg-adjusted pairwise Gray’s tests. The proportional hazards assumption was checked by Schoenfeld residuals. The declining-genus score was also tested as a continuous predictor, alone and alongside CST. Community state and bacterial burden were also modeled jointly, entering CST and bacterial-burden tertiles together in the Cox model with a likelihood-ratio test for the added contribution of CST. To determine whether baseline CST reflects ecological susceptibility or proxies the eventual infecting organism, the association between baseline CST and the pathogen cultured at VAP onset was tested by Fisher’s exact test. For species-resolved VAP episodes, the cultured organism was also mapped to the genus level and matched to the corresponding baseline BAL 16S profile to assess baseline detection and abundance (Table S8).

#### Microbiome–cytokine associations

BAL cytokine concentrations (IL-1β, IL-6, and TNF-α) were compared across CSTs by Kruskal–Wallis tests followed by Dunn’s pairwise comparisons, with Benjamini–Hochberg adjustment within each cytokine. Each log-transformed cytokine was regressed on log-transformed bacterial burden, the declining-genus score, sex, and GCS, with overall *R*² and per-predictor coefficients reported. Collinearity between burden and the declining-genus score was assessed by Spearman correlation. VAP status was not included because it is a post-baseline event. In longitudinal models, a declining-genus score × timepoint interaction (LMM, patient random intercept) tested whether the association changed over time. To assess whether the microbiome–cytokine relationship depended on community state or changed over time, score × CST and score × timepoint interactions were tested by likelihood-ratio comparison; per-genus and per-cytokine CST-dependent trajectories were tested analogously.

#### Sensitivity analyses

Robustness of the CST–VAP association was assessed by (i) refitting DMM with *k* = 4 and *k* = 2 to test whether risk stratification was sensitive to model complexity; (ii) refitting DMM to all 132 baseline samples including early-extubation patients; (iii) adjusting longitudinal models for antibiotic administration within the first 24 h; and (iv) logistic regression as an alternative to the Cox framework.

## Supplementary Material

Reviewer comments

## Data Availability

Raw 16S rRNA gene sequencing reads analyzed in this study are publicly available in the NCBI Sequence Read Archive under BioProject accession number PRJNA1212098. Clinical metadata, inflammatory marker data, ddPCR measurements, and processed microbiome data are available through the PhysioNet MicroNAV data set v1.1.1 (https://doi.org/10.13026/tyj5-dd44). All analyses were performed in R v4.5.3. The complete R analysis workflow used for sequence processing, decontamination, statistical analyses, and generation of all tables and figures is available at https://github.com/zhihao-code/vap_microbiome_analysis_1.

## SUPPLEMENTAL MATERIAL

Supplemental tables and figure spectrum.01263-26-s0001.docx Tables S1 to S14 and Fig. S1.

OPEN PEER REVIEW reviewer-comments.pdf An accounting of the reviewer comments and feedback.

ASM does not own the copyrights to Supplemental Material that may be linked to, or accessed through, an article. The authors have granted ASM a non-exclusive, world-wide license to publish the Supplemental Material files. Please contact the corresponding author directly for reuse.
