# Supplementary material for: Lung microbiome community state types are associated with ventilator-associated pneumonia risk: a secondary analysis of the MicroNAV cohort
Source: Microbiol Spectr. 2026 Oct 8;14(11):e01263-26. doi: 10.1128/spectrum.01263-26 (PMC13729170; doi:10.1128/spectrum.01263-26)
Supplement: Supplementary file 1 [file reviewer-comments.pdf]

ASM | Microbiology Spectrum

Lung microbiome community state types are associated with ventilator-associated pneumonia risk: a secondary analysis of the MicroNAV cohort

Shiji Xiao, Qiufeng Zhuang, Guobin Cui, and Yinglin Li

Corresponding Author(s): Yinglin Li, Affiliated Hospital of Putian University

Review Timeline:

Submission Date:	April 13, 2026
Editorial Decision:	May 14, 2026
Revision Received:	June 26, 2026
Editorial Decision:	July 16, 2026
Revision Received:	August 13, 2026
Accepted:	August 14, 2026

Editor: Bo-young Hong

Reviewer(s): Disclosure of reviewer identity is with reference to reviewer comments included in decision letter(s). The following individuals involved in review of your submission have agreed to reveal their identity: Ahmed Adel Baz (Reviewer #2)

Transaction Report:

(Note: With the exception of the correction of typographical or spelling errors that could be a source of ambiguity, letters and reports are not edited. The original formatting of letters and referee reports may not be reflected in this compilation.)

DOI: https://doi.org/10.1128/spectrum.01263-26

Re: Spectrum01263-26 (**Lung microbiome community state types and commensal-immune coupling during mechanical ventilation in the MicroNAV cohort**)

Dear Dr. Yinglin Li:

Thank you for the privilege of reviewing your work. Below you will find my comments, instructions from the Spectrum editorial office, and the reviewer comments.

Please address the reviewer's thorough and constructive feedback to improve the manuscript.

Please return the manuscript within 60 days; if you cannot complete the modification within this time period, please contact me. If you do not wish to modify the manuscript and prefer to submit it to another journal, notify me immediately so that the manuscript may be formally withdrawn from consideration by Spectrum.

Revision Guidelines
To submit your modified manuscript, log into the submission site at https://spectrum.msubmit.net/cgi-bin/main.plex. Go to Author Tasks and click the appropriate manuscript title to begin. The information you entered when you first submitted the paper will be displayed; update this as necessary. Note the following requirements:

• Upload point-by-point responses to the issues raised by the reviewers in a file named "Response to Reviewers," NOT in your cover letter.
• Upload a compare copy of the manuscript (without figures) as a "Marked-Up Manuscript" file.
• Upload a clean .DOC/.DOCX version of the revised manuscript and remove the previous version.
• Each figure must be uploaded as a separate, editable, high-resolution file (TIFF or EPS preferred), and any multipanel figures must be assembled into one file.
• Any supplemental material intended for posting by ASM should be uploaded with their legends separate from the main manuscript. You can combine all supplemental material into one file (preferred) or split it into a maximum of 10 files with all associated legends included.

For complete guidelines on revision requirements, see our Submission and Review Process webpage. Submission of a paper that does not conform to guidelines may delay acceptance of your manuscript.

Data availability: ASM policy requires that data be available to the public upon online posting of the article, so please verify all links to sequence records, if present, and make sure that each number retrieves the full record of the data. If a new accession number is not linked or a link is broken, provide Spectrum production staff with the correct URL for the record. If the accession numbers for new data are not publicly accessible before the expected online posting of the article, publication may be delayed; please contact production staff (Spectrum@asmusa.org) immediately with the expected release date.

Publication Fees: For information on publication fees and which article types are subject to charges, visit our website. If your manuscript is accepted for publication and any fees apply, you will be contacted separately about payment during the production process; please follow the instructions in that e-mail. Arrangements for payment must be made before your article is published.

ASM Membership: Corresponding authors may join or renew ASM membership to obtain discounts on publication fees. Need to upgrade your membership level? Please contact Customer Service at Service@asmusa.org.

The ASM Journals program strives for constant improvement in our submission and publication process. Please tell us how we can improve your experience by taking this quick Author Survey.

Thank you for submitting your paper to Spectrum.

Sincerely,
Bo-young Hong
Editor
Microbiology Spectrum

Reviewer #1 (Comments for the Author):

I am reviewing the manuscript, "Lung microbiome community state types and commensal-immune coupling during mechanical ventilation in the MicroNAV cohort" submitted by Dr. Shiji et al. and collaborators. While the analysis is a re-evaluation of a published dataset and manuscript, the authors did not discuss any findings associated with the previous publication, which I feel is a disservice to the work - for example - how do the analyses compare? Moreover, the CST states, which as associated with

VAP and rates of VAP seem to be modest, approximately around 20%, and it's not clear that the signatures are discriminatory for VAP. Lastly, there is very little cohesive discussion in regards to the overall conclusions of the manuscript and what similarities and differences between the already been published manuscript based upon this data (Bustos et al.)

1. The main issue is that the manuscript reads similarly as a disconnected sub-analysis of a published manuscript, thus the conclusions of the manuscript seems disconnected and issues with the conclusion read similarly to a list of different results, rather than a conclusion. Overall, there are different interesting findings separately, but the analysis rarely refers to findings from the original Bustos manuscript. I think that the authors should consider reaching out to the original authors and discussing perhaps some analysis which would complement the primary manuscript.

2. Very little analyses on confounders, covariates, the authors likely have limited access to issues such as antibiotics, severity of disease, respiratory failure, and outcomes outside of VAP and mortality. These are missing for a more thorough understanding of the disease. Moreover, the creation of several outcomes, such as the 13-commensal grouping is not clearly explained - why are these justified? I think that the authors could overall do a slightly better job in explaining why they decided to examine these outcomes.

3. Lack of code for review, microbiome standards require the inclusion of code for review, not upon reasonable request. This is specifically due to the evaluation of how the conclusions were reached. Unfortunately, without the code available in this submission, I consider the manuscript to be incomplete. The authors should have it available and I am requesting it to review before final approval.

I will be using the combined PDF pages (actual) and the line numbers in the PDF.

Major.

Abstract.

Alter as needed

Introduction.

Page 4, Line 55 "VAP pathogenesis involves micro-aspiration of oropharyngeal and gastric contents," This needs a citation please.

Page 4, Line 57 "Despite the clinical burden of VAP, the ecological mechanisms by which the lung microbiome transitions from a commensal-dominated to a pathogen-dominated state remain incompletely understood." I know this is what the authors are seeking to study, but this is not completely discussed well to understand why the authors want to study this. Why do we care? It's not clear from the reasoning of why this should be the case.

Page 5, Line 75 "Several analytical opportunities remained from this foundational
work, including genus-level differential abundance testing with covariate adjustment, unsupervised community classification, and direct quantitative testing of microbiome-cytokine associations within the same samples." Have the authors considered working and/or working with the Bustos et al. group to see if there is any input in the findings of this manuscript?

Page 5, Line 79 "In this secondary analysis, we hypothesized that the baseline lung microbiome contains ecologically distinct community configurations with differential VAP incidence, and that commensal abundance is quantitatively coupled with local inflammatory tone." The authors do not really lead to this hypothesis very well? There should be some thorough discussion as to this hypothesis was formed, also it seems as though there are two different hypothesis here, local inflammatory tone and distinct community configurations - I think that the authors could d

Results.

Page 5, Line 90 "...223 observations..." And Page 5, Line 91 "135 Longitudinal observations" What are observations? Please explain a bit better here.

Page 6, Line 104 "...we applied unsupervised Dirichlet Multinomial Mixture (DMM) modelling to all 132 baseline samples..." Why not apply to all samples? I am wondering the the choice for baseline samples is interesting since it will only group the samples according to a timepoints, but that's assuming that the other groups do not impact the distribution of the DMM clusters.

Page 6, Line 106 "characterized by the highest abundance of oral commensals (Streptococcus, Prevotella, Veillonella, Haemophilus, Gemella, Neisseria), had a low VAP incidence (26%) " I am not sure if incidence is correctly used here, there the authors are describing known cases throughout the study, for a total group, which the time frame is known, nor are they describing a rate, this should be a prevalence of VAP. If they were talking about the rate that would develop, then incidence would be correct.

Page 6, Line 110 "intermediate community with reduced commensal representation, had the highest VAP rate (47%). CST3 (n = 33) harbored known VAP pathogens (Acinetobacter, Klebsiella, Staphylococcus) yet had a low VAP rate (24%)," The low rate VAP pathogens is not clear, it apparently has the lowest/highest rate occurred in CST3? Please correct as this sentence does not read clearly.

Page 7, Line 116 "...VAP patients in CST1 showed greater state persistence (64% vs. 35% in non-VAP), and the CST3-to-CST4 transition occurred in 60% of VAP but only 13% of non-VAP transitions, suggesting progressive ecological degradation accompanying VAP development (Table S1; Fig. S2)." The state persistence likely is explained during the methods, but the authors could do a slightly improved job to discuss this here - it would help understand this.

Page 7, Line 126 "Nine of the 13 declining genera ranked within the top 15 defining genera of CST2 (Fig. 2C), with component weights 2- to 8-fold higher than in other CSTs (Streptococcus: 12.6 in CST2 vs. 3.2 in CST1; Neisseria: 2.3 vs. 0.4), indicating that depletion disproportionately erodes the genera most abundant in the lower-VAP community configuration." This makes sense, but it's methodological, especially if CST and the ranked genera - the significance of the lost of these oral commensals are not sure that aside from the hypothesis this was important to re-analysis. I think that the authors could explain why these were important.

Page 7, Line 129 "None of the 13 showed a significant timepoint × VAP interaction (all FDR > 0.10; Table 3), confirming that commensal loss was a general consequence of ventilation rather than a VAP-specific process." The finding is consistent with Kitsios work and validated by other studies.

Page 8, Line 147 "To directly quantify this relationship, we constructed a 13-genus commensal score (sum of CLR-transformed abundances of the declining genera) and regressed it against baseline cytokine concentrations, adjusting for gender and GCS." Not sure if the authors actually justified this 13-genus commensal score? How did the authors determine this score? Are they associated with CSTs? This should be much better explained and why?

Discussion.

Page 9, Line 178 "Third, commensal abundance was quantitatively coupled with BAL inflammatory cytokines at baseline, and this coupling progressively weakened during ventilation, suggesting a role for commensals in maintaining tonic immune surveillance." I understand that the cytokines association was weakened, but saying that it is "linked" may even too strong. What about the sensitivity analysis for other confounders such as antibiotics, immunosuppressed, severity of respiratory failure/distress, length of intubation, etc.

Page 10, Line 187 The commensal-enriched CST2 had the lowest VAP incidence (OR = 0.34), consistent with the adapted island model in which communities dominated by oropharyngeal immigrants reflect intact mucosal defenses(5)." The problem with this line is that the original adapted island model did not address immune response or immune readiness/tone to respond to the lower airway infection. I think the authors make a logical leap here that needs to be connected much better either through experiments/citations of bench/translational information or work of Segal.

Page 10, Line 190 "Conversely, the pathogen-harboring CST3 had a low VAP rate despite elevated Acinetobacter, Klebsiella, and Staphylococcus abundance, and the lowest TNF-α among all CSTs -suggesting that pathogen presence does not inevitably lead to infection when immune surveillance remains quiescent. " The authors should also note that there could also be immunosuppression especially in critically ill participants.

Page 11, Line 214 "Under this interpretation, patients entering the ICU with higher commensal abundance (CST2) may tolerate greater ecological disruption before reaching a threshold permissive to infection, " I think that both higher commensal abundance is not entirely proven, perhaps saying that higher relative abundance of oral commensals (burden as defined by microbiome papers is the qPCR).

Page 11, Line 216 "The longitudinal CST tracking supports this: VAP patients in CST1 showed greater state persistence, suggesting entrainment in a high-risk configuration." I did not see how long - per days - that there is persistence in CST states.

Page 12, overall, the conclusion reads like a list of conclusions rather than an encapsulation of the re-interpretation of the paper, this lacks two specific issues, which the first is that the conclusion seems to just list their observations, yet do not quite extend and wrap up the study - instead the list of observations reads as though the authors are listing the results again. What does it mean for VAP? The other issue is the comparison and connection to the previous publication - what does it mean for Bustos' manuscript? I wish the authors compared their work to Bustos, I would walso consider reaching out to them as possible co-authors to help with possible missing elements of the manuscript.

Materials and Methods.

Page 13, Line 252 - I think the study design and data source should consider comparing the data published in Bustos to the

analysis they observed - although it it is a reanalysis, the authors should make clear that any of the primary collection of the data is not their work, as it reads, the repeating of the study itself seems too much as though it was included as part of the work - perhaps making it clear that the data was part of a primary study that the re-analysis / publication authors did not have any role in.

Page 14, Line 275 "Fifty-nine ASVs identified as contaminants were removed. Samples with fewer than 1,300 quality-filtered reads post-decontamination were excluded." Please include a justification of the contamination, did the authors use any of data such as decontamination (decontam) ?

Page 15, Line 288 "Model B" The authors only adjusted two particular gender and GCS, what about age, antibiotics, severity of disease, culture positivity/negativity? I think there is more adjustments that the authors could make here, especially complicated participants.

Page 15, Line 294 "covariates" which ones, the authors should discuss them here.

Page 16, Line 312 "Statistical software. All analyses were performed in R v4.3.1 using DADA2, decontam, DirichletMultinomial, DESeq2, MaAsLin3, lme4, lmerTest, vegan, and compositions. Code is available at https://github.com/datianren07-rgb/vap_microbiome_analysis (to be made public upon acceptance; available to reviewers upon request). " Please specify which versions were used for the analysis.

Page 16, Line 321 "Analysis code is available at https://github.com/datianren07-rgb/vap_microbiome_analysis (to be made public upon acceptance; available to reviewers upon request)." Please provide the code for review. The standard for all microbiome studies is that code is available for review, this is the standard for all microbiome studies.

Minor.

Just a re-read to make sure the logic flows well.

Reviewer #2 (Comments for the Author):

This is a well-designed secondary analysis of the MicroNAV cohort that addresses an important gap: linking lung microbiome community structure to ventilator-associated pneumonia (VAP) risk and local immune tone. The DMM community typing and commensal-immune coupling findings are novel and clinically meaningful. Major strengths include rigorous bioinformatics (DADA2 + decontam + ddPCR calibration), longitudinal modeling, and clear mechanistic framing. Belowe comments;
Lines 92-95: VAP patients were more frequently female (p=0.02) and had lower GCS... Gender and GCS were included as covariates in all adjusted models.
Suggestion: Justify covariate selection briefly. Given baseline imbalances in sex and neurological severity, gender and GCS were included as a priori clinical covariates (Model B) to minimize confounding.Report percentages.
Lines 108-109: CST2... OR=0.34, 95% CI 0.13-0.91, p=0.031. CST1... OR reference.
Only CST2 reports CI. All CSTs vs CST1 should report OR, CI, and p-value in text for transparency.
Suggestion: Add CIs for CST3 (0.15-1.14) and CST4 (0.09-1.31) in the results narrative.
Lines 115-117: CST3-to-CST4 transition occurred in 60% of VAP but only 13% of non-VAP transitions
Suggestion: Provide absolute N for transitions. ...occurred in 6/10 VAP transitions vs 2/15 non-VAP transitions (Table S1).
Lines 132-139: ddPCR-derived total bacterial DNA concentrations... Total bacterial burden remained stable over time (LMM p=0.80)
Ambiguity on whether ddPCR measured total DNA or 16S rRNA gene copies. Also, ddPCR was likely performed on a subset, not all 223 samples.
Suggestion: Clarify in methods and results: Absolute abundances were estimated by scaling relative abundances to ddPCR-quantified bacterial 16S rRNA gene copy numbers (copies/μL BAL). Calibration was performed on [N] representative samples; temporal stability of total bacterial burden was confirmed (LMM p=0.80).
Lines 155-160: p=0.053 and 0.072... approaching significance
"Approaching significance" is discouraged in modern statistical reporting.
Suggestion: Replace with showed directional trends consistent with attenuation (p=0.053 and p=0.072, respectively). Add caveat: Given reduced sample sizes at later timepoints (n=36, 21), these exploratory cross-sectional regressions should be interpreted descriptively.
Lines 162-170: Sensitivity analyses. Well-executed. Suggest adding a sentence on antibiotic confounding: Early antibiotic exposure did not alter the primary commensal depletion signature, supporting a ventilation-driven ecological shift rather than treatment effect.
Lines 219-228: Commensal-immune coupling paragraph.
Suggestion: Acknowledge that BAL cytokines may reflect non-microbial inflammation (e.g., ventilator-induced lung injury, aspiration). Add: While BAL cytokines correlate with commensal abundance, we cannot exclude that shared upstream factors (e.g., mucosal barrier integrity, ventilator settings) jointly drive both microbial composition and local inflammatory tone.
Lines 232-242: Limitations.

Add:
16S rRNA sequencing at the V4 region limits resolution to genus level and cannot capture strain-specific virulence factors or functional pathways.
BAL sampling captures distal airway microbiota but may miss proximal or regional heterogeneity.
Cross-sectional DMM typing assumes baseline states are discrete; transitional or gradient communities may exist.
Lines 278-283: DMM... posterior probability
Suggestion: State the threshold for CST assignment
Lines 296-297: Absolute genus-level abundances were estimated by multiplying relative abundances by ddPCR-derived total bacterial DNA concentrations
Suggestion: Change to bacterial 16S rRNA gene copy concentrations for accuracy. Note if host DNA was excluded or if universal bacterial primers were used.
Lines 289-294: timepoint as fixed effect
Suggestion: Clarify if timepoint was coded as continuous (hours/days) or categorical (1,2,3...). If categorical, state reference level.
Lines 299-302: CLR-transformed abundances
Suggestion: Specify zero-replacement method (e.g., CLR transformation after Bayesian-multiplicative zero replacement using zCompositions or similar). 16S data contains structural zeros; standard CLR fails without imputation.

This is a well-designed secondary analysis of the MicroNAV cohort that addresses an important gap: linking lung microbiome community structure to ventilator-associated pneumonia (VAP) risk and local immune tone. The DMM community typing and commensal–immune coupling findings are novel and clinically meaningful. Major strengths include rigorous bioinformatics (DADA2 + decontam + ddPCR calibration), longitudinal modeling, and clear mechanistic framing. Belowe comments;

- Lines 92–95: VAP patients were more frequently female (p=0.02) and had lower GCS... Gender and GCS were included as covariates in all adjusted models.

Suggestion: Justify covariate selection briefly. Given baseline imbalances in sex and neurological severity, gender and GCS were included as a priori clinical covariates (Model B) to minimize confounding.Report percentages.

- Lines 108–109: CST2... OR=0.34, 95% CI 0.13–0.91, p=0.031. CST1... OR reference.

Only CST2 reports CI. All CSTs vs CST1 should report OR, CI, and p-value in text for transparency.

Suggestion: Add CIs for CST3 (0.15–1.14) and CST4 (0.09–1.31) in the results narrative.

- Lines 115–117: CST3-to-CST4 transition occurred in 60% of VAP but only 13% of non-VAP transitions

Suggestion: Provide absolute N for transitions. ...occurred in 6/10 VAP transitions vs 2/15 non-VAP transitions (Table S1).

- Lines 132–139: ddPCR-derived total bacterial DNA concentrations... Total bacterial burden remained stable over time (LMM p=0.80)

Ambiguity on whether ddPCR measured total DNA or 16S rRNA gene copies. Also, ddPCR was likely performed on a subset, not all 223 samples.

Suggestion: Clarify in methods and results: Absolute abundances were estimated by scaling relative abundances to ddPCR-quantified bacterial 16S rRNA gene copy numbers (copies/μL BAL). Calibration was performed on [N] representative samples; temporal stability of total bacterial burden was confirmed (LMM p=0.80).

- Lines 155–160: p=0.053 and 0.072... approaching significance

"Approaching significance" is discouraged in modern statistical reporting.

Suggestion: Replace with showed directional trends consistent with attenuation (p=0.053 and

p=0.072, respectively). Add caveat: Given reduced sample sizes at later timepoints (n=36, 21), these exploratory cross-sectional regressions should be interpreted descriptively.

- Lines 162–170: Sensitivity analyses. Well-executed. Suggest adding a sentence on antibiotic confounding: Early antibiotic exposure did not alter the primary commensal depletion signature, supporting a ventilation-driven ecological shift rather than treatment effect.
- Lines 219–228: Commensal-immune coupling paragraph.

Suggestion: Acknowledge that BAL cytokines may reflect non-microbial inflammation (e.g., ventilator-induced lung injury, aspiration). Add: While BAL cytokines correlate with commensal abundance, we cannot exclude that shared upstream factors (e.g., mucosal barrier integrity, ventilator settings) jointly drive both microbial composition and local inflammatory tone.

- Lines 232–242: Limitations.

Add:

16S rRNA sequencing at the V4 region limits resolution to genus level and cannot capture strain-specific virulence factors or functional pathways.

BAL sampling captures distal airway microbiota but may miss proximal or regional heterogeneity.

Cross-sectional DMM typing assumes baseline states are discrete; transitional or gradient communities may exist.

- Lines 278–283: DMM... posterior probability

Suggestion: State the threshold for CST assignment

- Lines 296–297: Absolute genus-level abundances were estimated by multiplying relative abundances by ddPCR-derived total bacterial DNA concentrations

Suggestion: Change to bacterial 16S rRNA gene copy concentrations for accuracy. Note if host DNA was excluded or if universal bacterial primers were used.

- Lines 289–294: timepoint as fixed effect

Suggestion: Clarify if timepoint was coded as continuous (hours/days) or categorical (1,2,3...). If categorical, state reference level.

- Lines 299–302: CLR-transformed abundances

Suggestion: Specify zero-replacement method (e.g., CLR transformation after Bayesian-multiplicative zero replacement using zCompositions or similar). 16S data contains structural zeros; standard CLR fails without imputation.

Response to Reviewers

Manuscript ID: Spectrum01263-26

Original title: Lung microbiome community state types and commensal-immune coupling during mechanical ventilation in the MicroNAV cohort

Revised title: Lung microbiome community state types are associated with ventilator-associated pneumonia risk: a secondary analysis of the MicroNAV cohort

We are grateful to the Editor and both Reviewers for their careful, rigorous, and constructive evaluation of our manuscript. We particularly appreciate the detailed methodological and conceptual comments, which were instructive for us and helped us improve the analytical framework, clarify the relationship between this secondary analysis and the parent MicroNAV study, and refine the treatment of methodological and interpretive limitations. We have taken these comments seriously and have substantially revised the manuscript in response.

Before the point-by-point responses, we would like to highlight one overarching revision that affects several comments and how they map onto the revised manuscript. During revision, we refined the central focus of the study and retitled the manuscript from "Lung microbiome community state types and commensal-immune coupling during mechanical ventilation in the MicroNAV cohort" to "Lung microbiome community state types are associated with ventilator-associated pneumonia risk: a secondary analysis of the MicroNAV cohort." This change reflects the revised emphasis of the manuscript: the primary finding is now presented as the association between baseline lung microbiome community state types and subsequent VAP risk, rather than as a commensal–immune coupling model.

We also clarified the analytical denominators and rebuilt the primary clinical risk analysis around the at-risk cohort of 75 mechanically ventilated patients with paired longitudinal BAL sampling. This cohort was distinguished from the broader quality-controlled baseline microbiome cohort and from assay-specific subsets used for cytokine, ddPCR, longitudinal microbiome, and survival analyses. Where event timing was available, we replaced occurrence-only comparisons with competing-risk time-to-event analyses, using cause-specific Cox regression and cumulative incidence functions with extubation and death on ventilation incorporated in the competing-risk framework.

As a result, several numerical results and some line-anchored reviewer comments now correspond to revised analyses and revised text. Specifically, VAP frequency is now reported within the at-risk cohort, DMM modelling now resolves three community state types rather than four, VAP risk is estimated using time-to-event analyses, and ddPCR-derived bacterial burden is integrated more

explicitly to separate community-state associations from bacterial quantity. We state these changes explicitly in the relevant responses below. We believe that the reviewers' comments have substantially improved the rigor, clarity, and interpretation of the manuscript.

Overview of the Revised Analysis

To make the revised analysis easier to follow, we briefly summarize the logic of the current manuscript before addressing each comment.

1. We reprocessed the 16S rRNA sequencing data using the updated DADA2/SILVA workflow and implemented a clarified decontamination and quality-control pipeline.
2. We define the clinically relevant at-risk cohort as 75 mechanically ventilated patients with paired longitudinal BAL sampling, including 40 VAP and 35 non-VAP patients.
3. We show that standard baseline microbiome comparisons do not distinguish future VAP from non-VAP: no genus-level feature remained significant after adjustment for gender and GCS.
4. We classify baseline BAL communities using unsupervised DMM modelling, identifying three community state types with distinct ecological profiles, bacterial burdens, and subsequent VAP rates.
5. We test clinical relevance using time-to-VAP analyses. Cause-specific Cox regression and cumulative-incidence analyses, accounting for death during ventilation and extubation as competing events, show that CST-associated VAP risk is not explained by bacterial burden alone.
6. We analyze longitudinal dynamics during ventilation. Oral commensal-associated genera decline and non-oral taxa expand, while total bacterial burden remains stable; this restructuring occurs in both VAP and non-VAP patients.
7. Finally, we separate microbiome composition from bacterial quantity in cytokine models. Baseline IL-1β and TNF-α are explained primarily by bacterial burden rather than by the declining-genus score, supporting a more cautious interpretation of microbiome-inflammation associations.

The point-by-point responses below describe where each reviewer concern was addressed in the revised manuscript.

All page and line references below refer to the reviewers' original comments; corresponding changes are incorporated in the revised manuscript and marked-up manuscript.

Reviewer #1

General assessment

Comment: The manuscript is a re-evaluation of a published dataset and did not sufficiently discuss how the analyses compare with the previously published MicroNAV study by Bustos et al. The CST states associated with VAP and the VAP rates seemed modest, and the conclusions read like a list of results rather than a cohesive interpretation.

Response: We have addressed these concerns in the revised manuscript. First, the apparently modest CST-associated VAP rates resulted from the denominator issue described above. After restricting the analysis to the 75-patient at-risk cohort and applying competing-risk time-to-event analysis, the CSTs separated more clearly: VAP rates were 64% in CST1, 58% in CST3, and 35% in CST2; CST1 was associated with higher VAP hazard than CST2 in cause-specific Cox regression (HR 2.85, 95% CI 1.15–7.02, P = 0.023), and the cumulative incidence curves separated by Gray's test (P = 0.042). Second, we have added explicit comparison with the parent MicroNAV study in the Introduction and Discussion, explaining how the present analysis extends the original findings. Third, the Discussion and Conclusion have been rewritten to present an integrated interpretation: baseline lower-airway ecological state carries VAP-susceptibility information that is not captured by single taxa, diversity, bacterial burden alone, or the subsequent ventilation-associated trajectory.

Major points

Comment: 1. The manuscript reads as a disconnected sub-analysis of a published manuscript, and the conclusions read like a list of results. The authors should consider reaching out to the original Bustos et al. group.

Response: We agree that the original submission did not adequately position our work relative to the parent MicroNAV study. The revised manuscript now addresses this in three ways. In the Introduction, we explicitly summarize the parent study's findings and then state the analytical gaps that motivated our secondary analysis: community-state typing, competing-risk survival analysis, ddPCR-calibrated absolute abundance, and separation of bacterial burden from community composition in cytokine models. In the Discussion, we now directly compare our findings with the parent MicroNAV study and explain that baseline community-state analysis revealed microbiome-derived risk information not captured by single-taxon or diversity analyses. The Conclusion has also been rewritten as an integrated synthesis rather than a list of separate observations. This remains an independent secondary analysis of fully de-identified, publicly available data.

Comment: 2. There is very little analysis of confounders and covariates. The creation of several outcomes, such as the 13-commensal grouping, is not clearly explained.

Response: We have expanded both the covariate analysis and the explanation of the genus grouping. For confounding, the primary adjusted models include gender and GCS because these were the two baseline variables that differed between VAP and non-VAP groups. We now also report sensitivity models adjusting for age, SOFA, PaO_2/FiO_2 ratio, antibiotic exposure within 24 hours, and log-transformed bacterial burden, entered individually and in a combined model where appropriate. The original "commensal score" has been reframed as a data-driven declining-genus score. It is no longer presented as an arbitrary or prespecified commensal grouping. The score comprises the twelve genera that showed concordant decline during ventilation by both MaAsLin3 and confirmatory linear mixed models, using the same direction of effect and multiple-testing criteria. We then used this score to test whether the aggregate magnitude of the ventilation-associated declining-genus signal was associated with VAP risk or explained cytokine variation. The score did not independently predict VAP and did not explain the CST-associated risk signal, which is now stated explicitly. Its derivation and interpretation are described in Methods and Results.

Comment: 3. Code was not available for review. Microbiome standards require inclusion of code for review, not only upon reasonable request.

Response: We agree. The complete analysis code is now publicly available at https://github.com/zhihao-code/vap_microbiome_analysis_1. The repository includes the workflow for sequence processing, decontamination, statistical analyses, and figure/table generation. The Methods and Data and Code Availability sections have been updated accordingly. The previous "available upon request" wording has been removed.

Abstract

Comment: Alter as needed.

Response: The Abstract has been rewritten to reflect the corrected at-risk cohort, the three-CST DMM solution, the competing-risk survival results, the integration of bacterial burden, and the revised cytokine interpretation.

Introduction

Comment: Page 4, Line 55: "VAP pathogenesis involves micro-aspiration of oropharyngeal and gastric contents." This needs a citation.

Response: A citation has been added to support this statement. The revised sentence cites a review covering micro-aspiration and related mechanisms in VAP pathogenesis.

Comment: Page 4, Line 57: The motivation for studying ecological mechanisms is not clear. Why do we care?

Response: We have revised the Introduction to motivate the question more clearly. The revised text explains that VAP susceptibility is unlikely to be determined simply by whether a potential pathogen is already present at intubation, because only a subset of colonized patients develop clinical pneumonia. We therefore frame the relevant signal as potentially residing in the broader lower-airway ecosystem, where community composition, bacterial burden, and host inflammatory tone are jointly shaped. The Introduction also explains why relative-abundance data are difficult to interpret in low-biomass respiratory samples without quantitative bacterial burden, and why community-level structure may contain information not captured by diversity or single-genus comparisons.

Comment: Page 5, Line 75: "Several analytical opportunities remained from this foundational work..." Have the authors considered working with the Bustos et al. group?

Response: The revised Introduction now frames the present work as an independent secondary analysis that builds on and extends the parent MicroNAV study. The Methods section states explicitly that the reanalysis was conducted independently and that the original investigators had no role in the design, analysis, or interpretation of the present study. We also acknowledge the original investigators for depositing the sequencing data and associated clinical, inflammatory marker, and microbiome data that made this secondary analysis possible.

Comment: Page 5, Line 79: The hypothesis is not led into well and appears to contain two different hypotheses.

Response: We agree. The revised Introduction separates the original statement into two related but distinct questions. The first is the main hypothesis: that mechanically ventilated patients without pulmonary infection at baseline harbor distinct lower-airway community states with different subsequent VAP susceptibility. This is motivated by the adapted island model, the low-biomass nature of lower-airway samples, and prior evidence that respiratory microbiome composition or bacterial burden can carry clinical information. The second is an exploratory analytical question: whether baseline airway cytokine variation is more closely associated with bacterial burden or with community composition.

Results

Comment: Page 5, Lines 90–91: "223 observations" and "135 longitudinal observations." What are observations?

Response: We have replaced ambiguous use of "observations" with more precise terminology. The revised Results now state that 132 patients provided quality-controlled baseline BAL samples and that 75 patients with paired longitudinal sampling constituted the at-risk cohort. Because assay availability and valid event-time data differed across analyses, Table S1 now defines the denominator, analysis unit, event coding, and assay availability for each analysis.

Comment: Page 6, Line 104: DMM was applied to all 132 baseline samples. Why not apply it to all samples?

Response: In the revised analysis, DMM is applied to baseline BAL samples from the at-risk cohort rather than to all 132 baseline samples or to all timepoints. This change follows from the corrected denominator and the scientific question. The primary question concerns pre-infection lower-airway ecological states associated with subsequent VAP risk; therefore, baseline samples are the relevant input. Mixing baseline and follow-up samples would conflate the initial community state with ventilation-induced restructuring, which is analyzed separately. As a sensitivity analysis, we also re-derived CSTs in the full 132-patient baseline cohort. This analysis was directionally consistent with the primary at-risk analysis, but the effect estimates were attenuated and did not retain the same statistical strength, supporting the at-risk cohort as the more appropriate denominator for the primary VAP-risk analysis.

Comment: Page 6, Line 106: "Incidence" may not be the correct term; this should be prevalence or rate of VAP.

Response: We agree. The revised manuscript no longer uses "incidence" to describe simple within-CST event proportions. We now use "VAP rate" for the proportion of patients with VAP within each CST in the at-risk cohort, and reserve "cumulative incidence" for the Aalen–Johansen competing-risk estimates. The corrected VAP rates are 64% in CST1, 58% in CST3, and 35% in CST2.

Comment: Page 6, Line 110: The sentence describing the CST with VAP pathogens but low VAP rate does not read clearly.

Response: The original four-CST description has been replaced by the corrected three-CST framework. The revised Results now state the apparent paradox more clearly: CST2 had the lowest VAP rate despite a higher relative abundance of genera commonly associated with nosocomial

infection. We explain that, in low-biomass samples, high relative abundance of pathogen-associated taxa can reflect low competing biomass rather than high absolute pathogen load. This point is now made explicitly in both Results and Discussion and supports the need for ddPCR-calibrated interpretation.

Comment: Page 7, Line 116: State persistence should be better explained.

Response: The original state-persistence analysis belonged to the previous four-CST framework and has been replaced by a more conservative approach. Although follow-up samples can be projected onto the baseline-trained DMM model, ventilation substantially alters community composition, making specific CST transitions difficult to interpret biologically. We therefore no longer emphasize state persistence or individual transition patterns. Instead, the revised analysis uses PERMANOVA on Bray–Curtis dissimilarities to assess whether baseline CST structure remains evident after ventilation. The CSTs were compositionally distinct at baseline, but this structure was markedly weakened by the first follow-up, supporting a distinction between baseline risk-associated community states and subsequent ventilation-associated restructuring.

Comment: Page 7, Line 126: Please explain why the declining genera were important.

Response: We have clarified why the declining genera were analyzed in relation to the parent study. The parent MicroNAV analysis showed that BAL microbiome composition changed over time in both VAP and non-VAP patients, suggesting an effect of mechanical ventilation. In the revised manuscript, we extended this observation by asking which genus-level trajectories drove the temporal shift, whether these trajectories differed by subsequent VAP status, and whether they accounted for the baseline CST-associated risk signal. Using a dual-confirmation framework, we identified twelve predominantly oral commensal-associated genera that declined during ventilation while total bacterial burden remained stable. However, no genus showed a significant time-by-VAP interaction after multiple-testing correction, and the declining-genus score did not predict VAP or explain the CST-associated risk signal. We therefore interpret these declines as a shared ventilation-associated restructuring process rather than a VAP-specific mechanism.

Comment: Page 7, Line 129: The finding that none of the declining genera showed a significant timepoint × VAP interaction is consistent with Kitsios's work and other studies.

Response: We agree and have incorporated this interpretation cautiously in the Discussion. We now cite prior intubated or mechanically ventilated cohorts describing respiratory microbiome restructuring and interpret our findings as a shared ventilation-associated trajectory rather than a VAP-specific

signal. Because the cited studies differ in population and endpoints, we do not frame this as a direct replication of Kitsios's work.

Comment: Page 8, Line 147: The 13-genus commensal score needs justification. How was it determined? Is it associated with CSTs?

Response: The score has been revised and justified more transparently. It is now a declining-genus score composed of the twelve genera that met a dual-confirmation criterion: MaAsLin3 $q < 0.05$ and LMM FDR < 0.05 in the same direction. This score was not used as a clustering input and was derived independently from DMM. It naturally differs across CSTs because CSTs vary in baseline community composition, but it is used analytically to test whether the aggregate declining-genus signal predicts VAP or explains cytokine variation. It did not independently predict VAP, and baseline IL-1β and TNF-α were explained primarily by bacterial burden rather than by the declining-genus score. The revised analysis was highly concordant with the original result.

Discussion

Comment: Page 9, Line 178: Saying that commensal abundance is "linked" or "coupled" with cytokines may be too strong. What about sensitivity analyses for antibiotics, immunosuppression, severity of respiratory failure, length of intubation, and other confounders?

Response: We agree and have softened the language throughout. The revised manuscript no longer centers the interpretation on "commensal–immune coupling." Instead, it states that baseline IL-1β and TNF-α were more closely associated with bacterial quantity than with community composition. We also added an explicit caveat that shared upstream factors, including mucosal barrier integrity, aspiration burden, and ventilator settings, may jointly influence microbial composition and local cytokine concentrations. Sensitivity analyses were added for age, SOFA, PaO_2/FiO_2 ratio, early antibiotic exposure, and bacterial burden in the CST–VAP models, and residual confounding by antibiotic exposure, immunosuppression, aspiration burden, and other unmeasured factors is now acknowledged in the Limitations.

Comment: Page 10, Line 187: The adapted island model did not address immune readiness; this is a logical leap that needs better support.

Response: We have removed the implication that the adapted island model directly implies immune readiness. CSTs are now framed as ecological phenotypes shaped by microbial immigration, elimination, local growth conditions, and host defense. The revised Discussion uses the adapted island model to interpret community assembly, while citing translational work separately where discussing

oral enrichment and inflammatory tone. We avoid making claims about immune readiness that exceed the data.

Comment: Page 10, Line 190: The statement that pathogen presence does not inevitably lead to infection when immune surveillance remains quiescent should also consider immunosuppression.

Response: The revised Discussion no longer uses the phrase "immune surveillance remains quiescent." Instead, the apparent paradox is interpreted through quantitative calibration: high relative abundance of pathogen-associated taxa in a low-biomass sample may not indicate true pathogen overgrowth. The Limitations section now acknowledges that immunosuppression and other unmeasured host factors may influence both the airway microbiome and VAP risk.

Comment: Page 11, Line 214: Higher commensal abundance is not fully proven; consider saying higher relative abundance of oral commensals, because burden is defined by qPCR/ddPCR.

Response: We agree. The revised manuscript distinguishes compositional abundance from ddPCR-calibrated bacterial quantity. "Bacterial burden" refers to ddPCR-derived 16S rRNA gene copy concentrations, whereas community-composition features are described as relative abundance, genus proportions, or CLR-transformed scores. When genus-level absolute abundance is analyzed, it is explicitly described as ddPCR-calibrated. The prior wording implying unqualified "higher commensal abundance" has therefore been removed or reframed.

Comment: Page 11, Line 216: The duration of CST persistence is unclear.

Response: We agree. The original Discussion over-interpreted the CST transition analysis by implying persistence of a high-risk state without clearly defining its duration. In the revised manuscript, we no longer emphasize state persistence or individual CST transitions. Instead, we distinguish the baseline CSTs, which capture the pre-infection risk-associated community state, from subsequent ventilation-associated restructuring. This avoids inferring persistence beyond what the longitudinal data can support.

Comment: Page 12, overall: The conclusion reads like a list rather than an encapsulation. What does it mean for VAP, and how does it relate to the Bustos et al. publication?

Response: The Conclusion has been rewritten as an integrative statement. It now states that baseline BAL communities in mechanically ventilated patients resolved into ecological states with different subsequent VAP risk; that this signal was not captured by individual genera, pathogen-associated relative abundance, bacterial burden alone, or the microbial trajectory after ventilation; and that VAP

susceptibility may arise from heterogeneous host–microbial airway terrains best interpreted using both community structure and absolute bacterial burden. The Discussion also now explicitly explains how our findings extend the parent MicroNAV study: the absence of baseline diversity or single-genus differences in the parent study does not imply absence of microbiome-derived risk information, which becomes visible when baseline communities are treated as burden-calibrated ecological states.

Materials and Methods

Comment: Page 13, Line 252: The study design and data source should make clear that primary data collection was not the authors' work.

Response: We have revised the Methods to state clearly that this is a secondary analysis of publicly available data from the MicroNAV prospective observational cohort. The Methods now specifies the BioProject and PhysioNet data sources, summarizes the parent study design, and states that the reanalysis was conducted independently and that the original investigators had no role in the design, analysis, or interpretation of the present study.

Comment: Page 14, Line 275: Please justify the contamination handling. Did the authors use decontam?

Response: Yes. The revised Methods now describe the decontamination procedure, and the supplementary material provides the corresponding ASV-level and pipeline summaries. Contamination was assessed using decontam with the combined frequency–prevalence method, incorporating ddPCR-derived bacterial 16S rRNA gene copy concentrations and extraction/isolation negative controls. Forty-six ASVs were identified as contaminants and removed; these are listed in Table S7. Table S8 summarizes the sequencing and bioinformatic pipeline, including removal of contaminant ASVs, mitochondrial/chloroplast ASVs, and samples below the 1,300-read threshold.

Comment: Page 15, Line 288: Model B adjusts only for gender and GCS. What about age, antibiotics, severity of disease, respiratory failure, and culture positivity/negativity?

Response: Gender and GCS were retained as the primary adjusted covariates because they were the variables imbalanced between VAP and non-VAP groups at baseline. We then added sensitivity models for the CST–VAP analysis adjusting for age, SOFA, PaO_2/FiO_2 ratio, early antibiotic exposure within 24 hours, and log-transformed bacterial burden, entered individually and in a combined model. Culture results are post-baseline features at VAP onset and therefore were not included as baseline covariates; instead, we analyzed whether the pathogen cultured at VAP onset differed by baseline CST and found no evidence of such an association.

Comment: Page 15, Line 294: The authors should specify which covariates were used.

Response: The revised Methods now explicitly lists covariates for each model type. Baseline differential abundance models include unadjusted and gender/GCS-adjusted models. CST–VAP survival models include gender and GCS in the primary model, with sensitivity models including age, SOFA, PaO_2/FiO_2 ratio, early antibiotic exposure, and log-transformed bacterial burden. Microbiome–cytokine models include bacterial burden, the declining-genus score, gender, and GCS.

Comment: Page 16, Line 312: Please specify which software and package versions were used.

Response: The revised Methods and Data and Code Availability section now specify the key software environment, including R, DADA2, and SILVA versions, and exact package versions are provided in sessionInfo.txt in the public code repository.

Comment: Page 16, Line 321: Please provide the code for review.

Response: The complete code is now publicly available at https://github.com/zhihao-code/vap_microbiome_analysis_1. The "available upon request" wording has been removed.

Minor comment

Comment: Please re-read to make sure the logic flows well.

Response: We have re-read and restructured the manuscript end to end. The revised narrative now follows a single argument: future VAP and non-VAP patients are not distinguished at baseline by standard diversity metrics or single-genus analyses; community-state typing reveals baseline ecological states with different VAP risk; this signal adds information beyond bacterial burden alone; mechanical ventilation produces shared microbiome restructuring that is not VAP-specific; and baseline IL-1β and TNF-α variation is explained primarily by bacterial burden. Together, these findings support the interpretation that baseline lower-airway ecological heterogeneity carries VAP-susceptibility information not captured by simpler microbiome summaries.

Reviewer #2

Overall comment

Comment: The reviewer considered the study a well-designed secondary analysis addressing an important gap and highlighted DMM community typing, ddPCR calibration, longitudinal modelling, and mechanistic framing as strengths.

Response: We thank the reviewer for this generous assessment and for the detailed, constructive suggestions. We have incorporated the suggestions throughout the revised manuscript, as detailed below.

Comment: Lines 92–95: Justify covariate selection briefly and report percentages.

Response: Adopted. The Results now state that VAP patients were more frequently female and had lower GCS scores at intubation, and that these two variables were therefore included as covariates in adjusted models. Detailed baseline characteristics and percentages are reported in the revised tables.

Comment: Lines 108–109: Report effect size, confidence interval, and P value for all CSTs versus the reference.

Response: Adopted, with the note that the revised analysis uses a corrected three-CST model and cause-specific Cox regression rather than the original four-CST logistic framework. The revised Results and Table 3 now report effect sizes, 95% confidence intervals, and P values for each non-reference CST: CST1 versus CST2, HR 2.85 (95% CI 1.15–7.02), $P = 0.023$; and CST3 versus CST2, HR 2.35 (95% CI 0.86–6.40), $P = 0.095$. CST2 was used as the reference because it had the lowest median bacterial burden and the lowest VAP rate.

Comment: Lines 115–117: Provide absolute N for transitions.

Response: The transition analysis belonged to the original four-CST framework and has been superseded by the corrected design. In the revised manuscript, baseline-versus-follow-up community structure is quantified by PERMANOVA rather than by emphasizing specific transition proportions. Table S1 now provides the analytic denominators, event counts, and assay availability for each analysis, so all reported proportions and model denominators are traceable to absolute sample sizes.

Comment: Lines 132–139: Clarify whether ddPCR measured total DNA or bacterial 16S rRNA gene copies; state the calibration N and confirm temporal stability.

Response: Adopted. The revised manuscript now consistently refers to ddPCR-derived bacterial 16S rRNA gene copy concentrations rather than total DNA. Absolute abundance was estimated by multiplying relative abundance by ddPCR-derived 16S rRNA gene copy concentration. We also state that ddPCR measurements were available only where sufficient material was available and provide assay availability in Table S1. Total bacterial burden remained stable over time in the at-risk cohort (LMM $P = 0.66$), supporting the interpretation that ventilation-associated changes reflect compositional restructuring rather than net bacterial clearance.

Comment: Lines 155–160: The phrase "approaching significance" is discouraged; replace it with exact P values and appropriate caveats.

Response: Adopted. The phrase "approaching significance" has been removed throughout the manuscript. The revised text reports exact P values and states that the longitudinal microbiome–cytokine interaction models had limited power because of reduced sample sizes at later timepoints. These exploratory longitudinal cytokine findings are therefore interpreted cautiously and descriptively.

Comment: Lines 162–170: Add a sentence on antibiotic confounding.

Response: Adopted. The Results now state that adjusting longitudinal models for early antibiotic exposure did not alter the set of dual-confirmed declining genera. Residual confounding by antibiotic exposure is also acknowledged in the Limitations.

Comment: Lines 219–228: Acknowledge that BAL cytokines may reflect non-microbial inflammation and that shared upstream factors may jointly drive composition and inflammatory tone.

Response: Adopted. The Discussion now states that BAL cytokines may partly reflect non-microbial inflammatory processes and that shared upstream factors, including mucosal barrier integrity, aspiration burden, or ventilator settings, may jointly influence both microbial composition and local cytokine concentrations. We also revised the interpretation of the cytokine models: baseline IL-1β and TNF-α were explained primarily by bacterial burden, whereas the declining-genus score was not independently associated with these cytokines after adjustment for burden.

Comment: Lines 232–242: Add limitations regarding 16S V4 resolution, BAL sampling, and DMM assumptions.

Response: Adopted. The Limitations now state that 16S rRNA V4 sequencing provides genus-level resolution and cannot assess strain-level virulence, antimicrobial resistance, or functional pathways; that BAL sampling captures the distal airway but may miss regional heterogeneity within the lung; and that DMM modelling imposes discrete states on what may be a continuum of ecological configurations.

Comment: Lines 278–283: State the posterior probability threshold for CST assignment.

Response: Adopted. The revised Methods now state that each sample was assigned to the CST with the highest posterior probability and that all assignment probabilities exceeded 0.90.

Comment: Lines 289–294: Clarify whether timepoint was coded as continuous or categorical.

Response: Adopted. The revised Methods now state that timepoint was entered as an integer-coded fixed effect corresponding to successive scheduled BAL sampling occasions and was treated as an ordered/continuous predictor in the longitudinal models.

Comment: Lines 296–297: Change "total bacterial DNA concentrations" to "bacterial 16S rRNA gene copy concentrations" and clarify host DNA exclusion.

Response: Adopted. The revised manuscript now uses "ddPCR-derived 16S rRNA gene copy concentrations" consistently. Host-derived mitochondrial and chloroplast sequences were removed bioinformatically, and absolute abundance estimates were based on bacterial 16S rRNA gene copy concentrations rather than total DNA.

Comment: Lines 299–302: Specify the zero-replacement method used before CLR transformation.

Response: Adopted. The revised Methods now specify that count zeros were replaced before CLR transformation using Bayesian-multiplicative replacement with zCompositions::cmultRepl, after restricting compositions to genera meeting the prevalence threshold. The previous pseudocount-based wording has been removed. Although this sensitivity analysis was not previously described, the main CLR-based results were also re-evaluated using pseudocount-based CLR transformation and remained qualitatively unchanged.

We again thank the Editor and Reviewers for their careful and constructive assessment of our manuscript. We hope that the revised analyses, additional methodological details, and clarified interpretation have adequately addressed the concerns raised.

Re: Spectrum01263-26R1 (**Lung microbiome community state types are associated with ventilator-associated pneumonia risk: a secondary analysis of the MicroNAV cohort**)

Dear Dr. Yinglin Li:

Thank you for the privilege of reviewing your work. Below you will find my comments, instructions from the Spectrum editorial office, and the reviewer comments.

Please address the reviewer's thorough and constructive feedback to improve the manuscript.

Please return the manuscript within 60 days; if you cannot complete the modification within this time period, please contact me. If you do not wish to modify the manuscript and prefer to submit it to another journal, notify me immediately so that the manuscript may be formally withdrawn from consideration by Spectrum.

Revision Guidelines
To submit your modified manuscript, log into the submission site at https://spectrum.msubmit.net/cgi-bin/main.plex. Go to Author Tasks and click the appropriate manuscript title to begin. The information you entered when you first submitted the paper will be displayed; update this as necessary. Note the following requirements:

• Upload point-by-point responses to the issues raised by the reviewers in a file named "Response to Reviewers," NOT in your cover letter.
• Upload a compare copy of the manuscript (without figures) as a "Marked-Up Manuscript" file.
• Upload a clean .DOC/.DOCX version of the revised manuscript and remove the previous version.
• Each figure must be uploaded as a separate, editable, high-resolution file (TIFF or EPS preferred), and any multipanel figures must be assembled into one file.
• Any supplemental material intended for posting by ASM should be uploaded with their legends separate from the main manuscript. You can combine all supplemental material into one file (preferred) or split it into a maximum of 10 files with all associated legends included.

For complete guidelines on revision requirements, see our Submission and Review Process webpage. Submission of a paper that does not conform to guidelines may delay acceptance of your manuscript.

Data availability: ASM policy requires that data be available to the public upon online posting of the article, so please verify all links to sequence records, if present, and make sure that each number retrieves the full record of the data. If a new accession number is not linked or a link is broken, provide Spectrum production staff with the correct URL for the record. If the accession numbers for new data are not publicly accessible before the expected online posting of the article, publication may be delayed; please contact production staff (Spectrum@asmusa.org) immediately with the expected release date.

Publication Fees: For information on publication fees and which article types are subject to charges, visit our website. If your manuscript is accepted for publication and any fees apply, you will be contacted separately about payment during the production process; please follow the instructions in that e-mail. Arrangements for payment must be made before your article is published.

ASM Membership: Corresponding authors may join or renew ASM membership to obtain discounts on publication fees. Need to upgrade your membership level? Please contact Customer Service at Service@asmusa.org.

The ASM Journals program strives for constant improvement in our submission and publication process. Please tell us how we can improve your experience by taking this quick Author Survey.

Thank you for submitting your paper to Spectrum.

Sincerely,
Bo-young Hong
Editor
Microbiology Spectrum

Reviewer #1 (Comments for the Author):

There are some missing pairwise analyses for figures 2D

pairwise p-values should be added onto figure panels Figure 2B, 2D, 2E
pairwise p-values should be added to figure 3C and 3B

pairwise p-values should be added to figure 4a
p-values should be added to 4B

Overall, the interpretation and rewriting of this manuscript is very interesting, I'm questioning and not quite sure if the interpretations are correct - while the community states seem to be associated with increase with VAP - the loss of certain taxa are significant with loss of particular taxa affecting CST1 and CST3 greater than CST2. Wouldn't analysis of this be not associated with say presence of oral commensal domination, but rather significant loss over time of oral commensals be associated with VAP?

Moreover, I want to see the groupings of oral commensals and pathogens - for example, staphylococcus which seems to be dominant in their group can be found as an oral taxon, I went through their manuscript but the authors do not clarify which taxa were included as oral taxon or pathogens. This hinders our understanding of Figure 2.

Lastly, the model that the authors discuss fit with a specific type of dysbiosis - which is connected to the adapted island model, but where the burden of aspiration and exposure to oral commensals may not be specifically helpful *(previous "healthy" patients were diagnosed with these oral commensals in the lower airway) - however, here the authors clearly connected evidence of increased aspiration (higher burden of bacterial load) + oral commensal CST signature is assocaited with VAP signatures. This is evident by greater inflammatory cytokines during the period of first sampling (baseline is 24 hours with respiratory failure). Suggesting that the authors have found a signature that is clearly associated with reasons of respiratory failure.

I am interested to understand why and which pathogens occurred in CST2 and did not develop VAP - the authors - if they can get antibiotic information would be important to understand its impact on the development on VAP. If this isn't available, it must be specified as a significant weakness of their interpretation. Moreover, it is also not clear how many people immediately received antibiotics upon intubation.

Moreover, it 's not clear how many patients are left to each group if 65% of CS1 developed VAP, I would want to know how many number of participants went into each group and if "pathogens" were identified by the authors resulted in pneumonia. I suspect that the reason why we're seeing the differences in a CST2 pathogen dominated group being the lowest is that these participants may have had culture data that lead to use of antibiotics more than the other two CST groups thus resulting in initial lower rates of VAP. If this information cannot be obtained it must be stated as a weakness. We found that the identification of pathogens in non-culture based techniques such as 16S resulted in culture positivity - thus - we may be observing the effects of antibiotic use highlighting the effect of significant aspiration -> identification of pathogen -> use of antibiotics -> lower overall VAP risk because participants were being treated for aspiration when they were intubated.

If AI was used to write the manuscript, the sentence structures seem to be unnecessarily complicated, this needs to be divulged.

Reviewer #2 (Comments for the Author):

Not more suggestions for authors

Response to Reviewers

Manuscript ID: Spectrum01263-26R1

Title: Lung microbiome community state types are associated with ventilator-associated pneumonia risk: a secondary analysis of the MicroNAV cohort

We thank the Editor and both Reviewers for their careful evaluation of our manuscript. We have revised the manuscript thoroughly in response to the comments and believe that these revisions have improved its clarity, methodological rigor, and interpretation. The reviewers' comments are reproduced below in italics, followed by our point-by-point responses. All corresponding changes have been incorporated into the revised manuscript and supplementary materials.

Reviewer #1

Comment 1 .*There are some missing pairwise analyses for figures 2D, pairwise p-values should be added onto figure panels Figure 2B, 2D, 2E, pairwise p-values should be added to figure 3C and 3B, pairwise p-values should be added to figure 4a, p-values should be added to 4B*

Response. We have added the requested comparisons to all relevant panels. Pairwise PERMANOVA was used for Fig. 2B, Dunn's tests for Figs. 2D and 4A, pairwise Gray's tests for Fig. 2E, paired Wilcoxon signed-rank tests for Fig. 3B, and pairwise slope contrasts from the baseline-CST × timepoint mixed-effects model for Fig. 3C. Pairwise P values were adjusted using the Benjamini–Hochberg method within each outcome, with all exact values reported in Table S4.

Overall, the additional analyses supported the patterns described in the manuscript: the strongest ecological, inflammatory, and VAP-risk differences generally

involved CST1 and CST2; bacterial burden did not differ significantly between longitudinal timepoints; and the CST-dependent decline in the declining-genus proportion was driven mainly by contrasts involving CST2. Figure 4B now reports the covariate-adjusted coefficients and P values from the joint multivariable models. These additions improve the statistical reporting of the figures but do not change the principal results or conclusions of the manuscript.

Comment 2. *the loss of certain taxa are significant with loss of particular taxa affecting CST1 and CST3 greater than CST2. Wouldn't analysis of this be not associated with presence of oral commensal domination, but rather significant loss over time of oral commensals be associated with VAP?*

Response. We agree that longitudinal commensal loss is an important alternative explanation, and we tested it directly. If loss rather than the baseline state accounted for VAP risk, patients who developed VAP would be expected to lose the declining-genus set more rapidly. We therefore assessed the timepoint × VAP interaction using three measures: the CLR score, the raw community proportion, and ddPCR-anchored estimated absolute abundance (Table S9).

The interaction was not significant for the CLR score (P = 0.66) or estimated absolute abundance (P = 0.27). The raw proportion showed a nominal interaction (P = 0.046), but this was not reproduced on the other two scales. Estimated absolute abundance of the declining-genus set decreased over time across the cohort (β = −0.71, P < 0.001), but the rate of decline did not differ by VAP status. At the individual-genus level, none of the 131 tested genera showed a significant timepoint

× VAP interaction after FDR correction. The baseline declining-genus score was also not associated with time to VAP (HR 1.13, 95% CI 0.78–1.61, $P = 0.52$); after baseline CST was added, the association remained non-significant (HR 0.63, 95% CI 0.36–1.08, $P = 0.09$).

The steeper decline observed in CST1 and CST3 addresses a different question, namely whether longitudinal change differs according to baseline community state. Genus-level CST × timepoint interactions were significant for 7 of the 12 declining genera (Table S10). At the aggregate level, the interaction was significant on the percentage-point scale ($P = 0.003$) but attenuated on the log scale ($P = 0.057$), indicating that the larger declines in CST1 and CST3 partly reflect their higher starting proportions. We therefore revised the Results and Discussion to describe commensal loss primarily as a general feature of mechanical ventilation rather than a reproducible VAP-specific trajectory.

Comment 3. *I want to see the groupings of oral commensals and pathogens — for example, staphylococcus which seems to be dominant in their group can be found as an oral taxon… the authors do not clarify which taxa were included as oral taxon or pathogens. This hinders our understanding of Figure 2.*

Response. We agree that these definitions were not sufficiently clear and have now made them explicit in Table S3.

The oral-associated set was derived directly from the eHOMD taxon table. Any genus whose Body Site annotation contained “Oral” and that was present in our data was included, without further manual curation. The respiratory pathogen-

associated set was a prespecified, non-exhaustive genus-level reference set based on the IDSA/ATS HAP/VAP framework and supporting respiratory microbiology literature. The included genera are listed in full in Table S3. The two sets were defined independently and were allowed to overlap. In our eHOMD-based annotation, Staphylococcus was classified as a nasal/skin rather than oral-associated genus and was therefore not included in the oral-associated set.

We recognize that V4 sequencing cannot distinguish pathogenic species from commensal congeners within several genera, including *Staphylococcus* and *Streptococcus*. This metric is therefore intended as a broad ecological summary of genera containing recognized respiratory pathogens or pathobionts. It should not be interpreted as pathogen burden or evidence of active infection. We have revised the terminology and clarified these definitions in the Methods and Table S3.

Comment 4. *here the authors clearly connected evidence of increased aspiration (higher burden of bacterial load) + oral commensal CST signature is assocaited with VAP signatures. This is evident by greater inflammatory cytokines during the period of first sampling (baseline is 24 hours with respiratory failure). Suggesting that the authors have found a signature that is clearly associated with reasons of respiratory failure.*

Response. We appreciate this interpretation and agree that CST1 may partly capture airway processes associated with the acute event leading to intubation, rather than a pre-existing ecological state that is wholly independent of respiratory failure. We have made this possibility explicit in the Discussion. Because baseline BAL was

collected within 12 hours of intubation, the high-burden, oral-associated, and inflammatory CST1 profile may reflect increased microbial immigration or impaired elimination during acute illness, including possible aspiration.

However, the available analyses suggest that CST1 is not simply a surrogate for measured neurologic or physiologic severity. The CST1–VAP association remained after adjustment for sex and GCS (HR 2.85, 95% CI 1.15–7.02, P = 0.023), and remained similar in models additionally adjusting for age, SOFA, and PaO_2/FiO_2. It also persisted after adjustment for bacterial burden, whereas bacterial-burden tertiles showed no graded association with VAP. Adding CST to the burden-based model improved model fit (P = 0.041).

The pairwise results further support an integrated ecological interpretation. CST1 and CST2 were the only pair that differed in both bacterial burden and oral-associated proportion, and this was also the only pair with a significant difference in cumulative VAP incidence (adjusted Gray's P = 0.025). CST1 versus CST3, which differed mainly in bacterial burden, and CST2 versus CST3, which differed mainly in oral-associated proportion, did not show significant differences in VAP incidence (P = 0.570 and 0.164, respectively). Although this pattern does not establish a formal interaction, it is consistent with the VAP association reflecting the broader community configuration rather than either feature considered in isolation.

We therefore revised the Discussion to describe CST1 as an early lower-airway ecological and inflammatory phenotype that may both reflect processes surrounding

acute respiratory failure and identify greater vulnerability to subsequent VAP. Because aspiration and airway protection were not measured directly, we cannot determine how much of this phenotype preceded the acute event or arose during it.

Comment 5. *I am interested to understand why and which pathogens occurred in CST2 and did not develop VAP - the authors - if they can get antibiotic information would be important to understand its impact on the development on VAP. If this isn't available, it must be specified as a significant weakness of their interpretation. Moreover, it is also not clear how many people immediately received antibiotics upon intubation. Moreover, it 's not clear how many patients are left to each group if 65% of CS1 developed VAP, I would want to know how many number of participants went into each group and if "pathogens" were identified by the authors resulted in pneumonia. I suspect that the reason why we're seeing the differences in a CST2 pathogen dominated group being the lowest is that these participants may have had culture data that lead to use of antibiotics more than the other two CST groups thus resulting in initial lower rates of VAP. If this information cannot be obtained it must be stated as a weakness. We found that the identification of pathogens in non-culture based techniques such as 16S resulted in culture positivity - thus - we may be observing the effects of antibiotic use highlighting the effect of significant aspiration -> identification of pathogen -> use of antibiotics -> lower overall VAP risk because participants were being treated for aspiration when they were intubated.*

Response. We appreciate this important alternative interpretation. CST2 included 23 patients, of whom 15 did not subsequently develop VAP. Among these 15 patients, baseline 16S detected *Escherichia-Shigella* in 13 patients, *Haemophilus* in 14, *Staphylococcus* in all 15, *Acinetobacter* in all 15, and *Klebsiella* in 14. Their mean relative abundances were 9.69%, 5.71%, 5.37%, 5.11%, and 2.39%, respectively. For comparison, the corresponding mean relative abundances among the eight CST2 patients who developed VAP were 0.23%, 4.66%, 3.58%, 2.16%, and 6.75%. Thus, these pathogen-associated genera were frequently detected in CST2 patients who remained free of VAP, and four of the five leading genera also had numerically higher mean relative abundances in this subgroup. These subgroup results are descriptive and were not subjected to formal hypothesis testing.

Relative abundance, however, does not indicate bacterial quantity. CST2 had the highest relative abundance of the pathogen-associated genus set (median 18.7%), but the lowest total bacterial burden (7.4×10^2 copies/μL) and the lowest ddPCR-anchored estimated absolute abundance of this set (1.9×10^2 copies/μL; Table S5). Thus, the apparent enrichment of these genera occurred within a low-biomass community. Accordingly, the higher relative abundance observed in CST2 should be interpreted in the context of its low bacterial burden.

The source dataset records antibiotic use only as a binary indicator of administration within the first 24 hours. Antibiotic use was recorded in 29 of 75 patients (39%) and did not differ across CSTs (CST1 42%, CST2 43%, CST3 26%; $P = 0.464$) or by subsequent VAP status (non-VAP 37% versus VAP 40%; $P = 0.82$). Adjustment

for this variable did not materially change the CST association (Model (Sex, GCS, Antibiotics within 24h), Table 3). Notably, CST1 and CST2 had nearly identical recorded antibiotic exposure (42% versus 43%) despite different VAP frequencies (64% versus 35%). The CST-specific denominators were already reported in Table 1 (CST1, 33 patients: 21 VAP and 12 non-VAP; CST2, 23: 8 and 15; CST3, 19: 11 and 8).

We also examined whether organisms detected by baseline 16S were subsequently recovered by culture at VAP onset. Among 39 VAP episodes with species-level identification, the genus later cultured was already detectable at baseline in 30 (77%). This was somewhat higher than the prevalence-matched expectation (30 versus 26.3; $P = 0.056$), although the small number of evaluable episodes limits this comparison. Baseline abundance showed a clearer signal, with a mean genus-specific percentile of 0.63 versus an expected 0.50 ($P = 0.006$). These results suggest some correspondence between the baseline microbiome and the organism later recovered by culture, but should be interpreted cautiously given the sample size. However, this baseline-to-culture correspondence did not appear to be driven by CST: baseline detection did not differ across CSTs ($P = 1.00$; Table S8), and baseline CST was associated with neither the Gram category ($P = 0.17$) nor the species identity ($P = 0.98$) cultured at VAP onset (Table S7).

These findings partly support the reviewer's observation that potential respiratory pathogens identified at baseline may later be recovered by culture. However, the available data do not show greater recorded antibiotic use in CST2, despite the

frequent presence of pathogen-associated genera in CST2 patients who remained free of VAP. Because antibiotic information was limited to a binary indicator for the first 24 hours, we cannot determine whether pathogen recognition led to targeted treatment or whether subsequent antibiotic management contributed to the lower VAP occurrence in CST2. Antibiotic class, indication, exact timing relative to intubation, duration, subsequent changes, and culture-directed therapy were unavailable. As suggested by the reviewer, we have added this limitation explicitly to the Discussion.

Comment 6. If AI was used to write the manuscript, the sentence structures seem to be unnecessarily complicated, this needs to be divulged.

Response. We appreciate this comment. The use of AI-assisted tools for English translation and language polishing was disclosed in the submission system at the time of submission. In response to the reviewer's concern, we have also reviewed the manuscript throughout and simplified unnecessarily complex sentence structures to improve clarity and readability.

Reviewer #2

No more suggestions for authors.

Response. We thank Reviewer #2 for the positive assessment and for the time taken to review our manuscript.

Re: Spectrum01263-26R2 (**Lung microbiome community state types are associated with ventilator-associated pneumonia risk: a secondary analysis of the MicroNAV cohort**)

Dear Dr. Yinglin Li:

Your manuscript has been accepted, and I am forwarding it to the ASM production staff for publication. Your paper will first be checked to make sure all elements meet the technical requirements. ASM staff will contact you if anything needs to be revised before copyediting and production can begin. Otherwise, you will be notified when your proofs are ready to be viewed.

Data Availability: ASM policy requires that data be available to the public upon online posting of the article, so please verify all links to sequence records, if present, and make sure that each number retrieves the full record of the data. If a new accession number is not linked or a link is broken, provide production staff with the correct URL for the record. If the accession numbers for new data are not publicly accessible before the expected online posting of the article, publication may be delayed; please contact ASM production staff immediately with the expected release date.

Publication Fees: For information on publication fees and which article types have charges, please visit ourwebsite. We have partnered with Copyright Clearance Center (CCC) to collect author charges. If fees apply to your paper, you will receive a message from no-reply@copyright.com with further instructions. For questions related to paying charges through RightsLink, please contact CCC at ASM_Support@copyright.com or toll free at +1-877-622-5543. CCC makes every attempt to respond to all emails within 24 hours.

ASM Membership: Corresponding authors may join or renew ASM membership to obtain discounts on publication fees. Need to upgrade your membership level? Please contact Customer Service at Service@asmusa.org.

Embargo Policy: A press release may be issued as soon as the manuscript is posted on theSpectrum Latest Articles webpage. The corresponding author will receive an email with the subject line "ASM Journals Author Services Notification" when the article is available online.

Cover Image Submissions: If you would like to submit a potential Cover Image, please email a file and a short legend to spectrum@asmusa.org. Please note that we can only consider images that (i) the authors created or own and (ii) have not been previously published. By submitting, you agree that the image can be used under the same terms as the published article. Image File requirements: TIF/EPS, 7.5 inches wide by 8.25 inches tall (at least 2,250 pixels wide by 2,475 pixels tall), minimum 300 dpi resolution (600 dpi preferred), RGB, and no figure elements, e.g., arrows or panel labels. The legend should be a short description of the image, 1-2 sentences recommended. Please use the ASM Journals Cover Image Sizing Tool (https://journals.asm.org/pb-assets/html/asm-cover-sizing-tool.html) to ensure that your proposed cover image meets our size requirements.

The ASM Journals program strives for constant improvement in our submission and publication process. Please tell us how we can improve your experience by taking this quick Author Survey.

Thank you for submitting your paper to Spectrum.

Sincerely,

Bo-young Hong
Editor
Microbiology Spectrum
