# Supplementary material for: Lung microbiome community state types are associated with ventilator-associated pneumonia risk: a secondary analysis of the MicroNAV cohort
Source: Microbiol Spectr. 2026 Oct 8;14(11):e01263-26. doi: 10.1128/spectrum.01263-26 (PMC13729170; doi:10.1128/spectrum.01263-26)
Supplement: Supplementary file 2 [file spectrum.01263-26-s0001.docx]

**Supplemental Material**

**Lung microbiome community state types are associated with ventilator-associated pneumonia risk: a secondary analysis of the MicroNAV cohort**

**Table S1. Analytic cohorts, event coding, and assay availability.**

This table defines the denominator used for each analysis. Counts differ because the unit of analysis varies across patient-level, baseline BAL sample-level, longitudinal BAL observation-level, and matched assay-level analyses.

| **Analysis** | **Unit / denominator** | **n used** | **VAP / non-VAP** | **Timepoints / events** | **Assay availability** | **Model-specific notes** |
| --- | --- | --- | --- | --- | --- | --- |
| MicroNAV cohort enrolled | Patients | 141 | 47 / 94 | — | — | Parent cohort. |
| Quality-controlled baseline BAL microbiome cohort | Baseline BAL samples | 132 | 44 / 88 | T1=132 | ddPCR: 126/132 | Sequencing QC passed. |
| Paired longitudinal at-risk cohort | Patients / baseline BAL | 75 patients; 75 baseline BAL | 40 / 35 | T1=75 | ddPCR: 71/75 | Primary clinical at-risk cohort. |
| CST identification and VAP occurrence | Baseline BAL samples | 75 | 40 / 35 | T1=75 | ddPCR: 71/75 | DMM k=3; CST1=33, CST2=23, CST3=19; |
| Cox / CIF VAP analysis | Patients | 74 | 40 / 34 | Within 14 days: 38 VAP events; death on ventilation = 6; extubation = 26; censored = 4 | ddPCR: 70/74 | One non-VAP patient was excluded because of missing time-to-event data; |
| Longitudinal microbiome analysis | BAL observations | 75 patients; 221 observations | 133 / 88 observations | T1=75 / T2=68 / T3=46 / T4=32 | ddPCR: 203/221 | MaAsLin3 + LMM. |
| Baseline cytokine-by-CST comparison | Baseline BAL samples | 70 | 39/31 | T1 =70 | Cytokines: 70/70 | KW test across CSTs (Fig. 4A). ddPCR not required. |
| Baseline microbiome-cytokine matched analysis | Matched baseline BAL samples | 68 | 38 / 30 | T1=68 | ddPCR: 68/68; cytokines: 68/68 | Joint regression on bacterial burden + declining-genus score (Table S11). |
| Longitudinal microbiome-cytokine matched analysis | Matched BAL observations | 74 patients; 138 observations | 73 / 65 observations | T1=70 / T2=37 / T3=23 / T4=8 | ddPCR: 129/138; cytokines: 138/138 | Matched longitudinal microbiome and cytokine observations. IL-1β=138, IL-6=138, TNF-α=138. |

Abbreviations: BAL, bronchoalveolar lavage; CIF, cumulative incidence function; CST, community state type; ddPCR, droplet digital PCR; DMM, Dirichlet multinomial mixture; LMM, linear mixed model; VAP, ventilator-associated pneumonia.

**Table S2. Clinical, microbiome, and inflammatory characteristics and outcomes of the at-risk cohort stratified by subsequent VAP status.**

| **Characteristic** | **Overall  N = 75^1^** | **Non-VAP  N = 35^1^** | **VAP  N = 40^1^** | ***P* value^2^** |
| --- | --- | --- | --- | --- |
| **Demographics** |  |  |  |  |
| Age, years | 51.0 (34.0–70.0) | 53.0 (31.0–70.0) | 49.0 (36.0–67.5) | 0.7 |
| Sex |  |  |  | **0.048** |
| Female | 24 (32%) | 7 (20%) | 17 (43%) |  |
| Male | 51 (68%) | 28 (80%) | 23 (58%) |  |
| BMI, kg/m² | 25.5 (23.5–27.7) | 25.0 (23.5–27.3) | 25.8 (23.6–28.5) | 0.5 |
| **Admission diagnoses** |  |  |  |  |
| Polytrauma | 32 (43%) | 15 (43%) | 17 (43%) | >0.9 |
| Post-operative (general/neurosurgery) | 27 (36%) | 9 (26%) | 18 (45%) | 0.10 |
| Intracranial hemorrhage | 22 (29%) | 7 (20%) | 15 (38%) | 0.13 |
| Hemorrhagic stroke | 7 (9.3%) | 5 (14%) | 2 (5.0%) | 0.2 |
| Ischemic stroke | 6 (8.0%) | 3 (8.6%) | 3 (7.5%) | >0.9 |
| Septic shock | 7 (9.3%) | 5 (14%) | 2 (5.0%) | 0.2 |
| Hypovolemic shock | 9 (12%) | 3 (8.6%) | 6 (15%) | 0.5 |
| **Comorbidities** |  |  |  |  |
| Arterial hypertension | 26 (35%) | 15 (43%) | 11 (28%) | 0.2 |
| Diabetes mellitus | 7 (9.3%) | 5 (14%) | 2 (5.0%) | 0.2 |
| Chronic kidney disease | 6 (8.0%) | 4 (11%) | 2 (5.0%) | 0.4 |
| **Severity scores at intubation** |  |  |  |  |
| GCS | 7 (6–10) | 8 (6–14) | 7 (6–8) | **0.04** |
| APACHE II | 16.5 (11–20) | 15 (9–19) | 17 (13–24) | 0.12 |
| SOFA | 8 (7–9) | 8 (6–9) | 8 (7–9) | 0.2 |
| CPIS | 2 (1–4) | 2 (1–5) | 2 (1–3) | 0.5 |
| SAPS | 2 (2–3) | 2.0 (2–3) | 2 (2–2) | 0.3 |
| **Vital signs at intubation** |  |  |  |  |
| Heart rate, bpm | 80.0 (66.0–98.0) | 90.0 (74.0–103.0) | 77.5 (58.5–94.0) | 0.059 |
| Respiratory rate, /min | 18.0 (16.0–20.0) | 18.0 (17.0–20.0) | 18.0 (16.0–20.0) | 0.7 |
| Temperature, °C | 36.5 (36.3–37.0) | 36.5 (36.2–36.8) | 36.6 (36.4–37.0) | 0.2 |
| SBP, mmHg | 120.0 (105.0–134.0) | 120.0 (101.0–140.0) | 120.5 (108.0–132.5) | 0.7 |
| MAP, mmHg | 102.7 (87.7–116.0) | 101.7 (85.3–125.0) | 103.0 (88.2–112.2) | 0.5 |
| SpO₂, % | 92.0 (91.0–95.0) | 93.0 (91.0–94.0) | 92.0 (91.5–95.5) | 0.6 |
| **Laboratory values at intubation** |  |  |  |  |
| WBC, ×10³/μL | 12.9 (9.5–16.3) | 11.7 (8.5–17.1) | 13.3 (11.2–16.1) | 0.3 |
| Neutrophils, % | 81.0 (71.0–87.0) | 81.0 (71.0–88.0) | 81.0 (71.0–87.0) | >0.9 |
| Hemoglobin, g/dL | 13.0 (11.4–14.0) | 12.3 (11.0–13.5) | 13.6 (11.7–14.6) | 0.10 |
| Platelets, ×10³/μL | 200.0 (160.0–270.0) | 200.0 (160.0–270.0) | 195.0 (155.0–275.0) | >0.9 |
| Creatinine, mg/dL | 1.0 (0.8–1.3) | 1.0 (0.8–1.3) | 1.0 (0.7–1.3) | 0.4 |
| BUN, mg/dL | 16.0 (12.0–22.0) | 17.0 (13.0–29.0) | 15.0 (12.0–20.0) | 0.2 |
| Glucose, mg/dL | 135.0 (120.0–160.0) | 140.0 (120.0–170.0) | 130.0 (120.0–160.0) | 0.8 |
| Sodium, mEq/L | 140.0 (138.0–142.0) | 140.0 (138.0–142.0) | 140.0 (138.0–143.0) | >0.9 |
| Potassium, mEq/L | 4.0 (3.6–4.6) | 4.0 (3.7–4.6) | 4.0 (3.6–4.6) | 0.7 |
| Lactate, mmol/L | 2.0 (1.2–3.2) | 2.3 (1.5–3.2) | 1.5 (0.9–3.2) | 0.2 |
| pH | 7.4 (7.2–7.4) | 7.4 (7.2–7.4) | 7.3 (7.2–7.4) | 0.2 |
| PaO₂, mmHg | 78.0 (70.0–94.0) | 85.0 (74.0–108.0) | 76.0 (68.5–88.0) | **0.030** |
| PaCO₂, mmHg | 37.0 (33.0–43.0) | 36.0 (33.0–40.0) | 37.5 (33.0–45.0) | 0.2 |
| FiO₂, % | 35.0 (28.0–40.0) | 35.0 (28.0–45.0) | 31.0 (28.0–37.5) | 0.5 |
| HCO₃, mEq/L | 20.0 (17.0–23.0) | 20.0 (16.0–23.0) | 20.0 (17.5–23.0) | 0.6 |
| PaO₂/FiO₂ ratio | 243.3 (200.0–294.0) | 246.9 (188.6–308.6) | 241.4 (203.6–292.9) | 0.4 |
| PT, seconds | 11.0 (10.0–13.0) | 12.0 (11.0–14.0) | 11.0 (10.0–12.0) | 0.11 |
| PTT, seconds | 24.0 (23.0–27.0) | 24.5 (23.0–28.0) | 24.0 (22.0–26.0) | 0.5 |
| **Airway microbiome and inflammation** |  |  |  |  |
| Shannon diversity | 2.8 (2.5–3.1) | 2.8 (2.5–3.1) | 2.7 (2.5–3.0) | 0.4 |
| Bacterial burden (ddPCR), copies/μL | 5.0 × 10⁴ (4.5 × 10³–5.5 × 10⁵) | 2.1 × 10⁴ (2.4 × 10³–3.4 × 10⁵) | 1.3 × 10⁵ (6.0 × 10³–9.0 × 10⁵) | 0.2 |
| IL-1β, pg/mL | 738.2 (4.6–4,972.4) | 535.9 (4.3–5,742.0) | 852.5 (4.6–2,559.7) | 0.9 |
| IL-6, pg/mL | 14.5 (1.0–78.5) | 26.4 (1.7–76.5) | 11.4 (0.8–83.2) | 0.7 |
| TNF-α, pg/mL | 51.9 (3.5–131.9) | 31.2 (2.9–104.5) | 53.8 (3.5–135.5) | 0.8 |
| **Treatment and outcomes** |  |  |  |  |
| Antibiotics within 24 h | 29 (39%) | 13 (37%) | 16 (40%) | 0.8 |
| Delirium during ICU | 30 (40%) | 16 (46%) | 14 (35%) | 0.4 |
| Extubated | 56 (77%) | 26 (76%) | 30 (77%) | >0.9 |
| ICU mortality | 17 (23%) | 7 (20%) | 10 (25%) | 0.8 |
| In-hospital mortality | 22 (29%) | 10 (29%) | 12 (30%) | >0.9 |
| IMV duration, days | 8.0 (5.0–11.0) | 6.0 (5.0–9.0) | 10.0 (7.5–15.5) | **<0.001** |
| ICU LOS, days | 13.0 (8.0–18.0) | 11.0 (6.0–14.0) | 15.5 (11.0–25.5) | **<0.001** |

^1^ Continuous variables are presented as median (IQR), and categorical variables are presented as n (%). Percentages were calculated using non-missing denominators.

^2^ P values were calculated using Wilcoxon rank-sum tests for continuous variables and Fisher's exact tests for categorical variables. P values are unadjusted.

Abbreviations: APACHE II, Acute Physiology and Chronic Health Evaluation II; BMI, body mass index; CPIS, Clinical Pulmonary Infection Score; ddPCR, droplet digital PCR; FiO₂, fraction of inspired oxygen; GCS, Glasgow Coma Scale; HCO₃, bicarbonate; ICU, intensive care unit; IMV, invasive mechanical ventilation; IQR, interquartile range; LOS, length of stay; MAP, mean arterial pressure; SAPS, Simplified Acute Physiology Score; SBP, systolic blood pressure; SOFA, Sequential Organ Failure Assessment; SpO₂, peripheral oxygen saturation; TNF-α, tumor necrosis factor alpha; VAP, ventilator-associated pneumonia; WBC, white blood cell count.

**Table S3. Classification and baseline abundance of pathogen-associated genera and major oral-associated genera.**

| **Genus** | **Oral-associated** | **Pathogen-associated** | **Prevalence (%)** | **Mean relative abundance (%)** |
| --- | --- | --- | --- | --- |
| **Assigned to both sets** | | | | |
| *Haemophilus* | Yes | Yes | 98.7 | 5.93 |
| *Serratia* | Yes | Yes | 1.3 | <0.01 |
| **Pathogen-associated only** | | | | |
| *Staphylococcus* | No | Yes | 90.7 | 2.60 |
| *Escherichia-Shigella* | No | Yes | 54.7 | 2.42 |
| *Acinetobacter* | No | Yes | 62.7 | 1.51 |
| *Klebsiella* | No | Yes | 60.0 | 1.37 |
| *Mycoplasma* | No | Yes | 69.3 | 0.70 |
| *Pseudomonas* | No | Yes | 45.3 | 0.37 |
| *Stenotrophomonas* | No | Yes | 9.3 | 0.02 |
| *Enterobacter* | No | Yes | 0.0 | 0.00 |
| **Major oral-associated genera only** | | | | |
| *Prevotella* | Yes | No | 100.0 | 15.11 |
| *Streptococcus* | Yes | No | 100.0 | 13.81 |
| *Veillonella* | Yes | No | 100.0 | 12.14 |
| *Fusobacterium* | Yes | No | 96.0 | 3.04 |
| *Segatella* | Yes | No | 98.7 | 2.94 |
| *Hoylesella* | Yes | No | 96.0 | 2.75 |
| *Neisseria* | Yes | No | 88.0 | 2.69 |
| *Gemella* | Yes | No | 98.7 | 2.40 |
| *Alloprevotella* | Yes | No | 96.0 | 2.00 |
| *Porphyromonas* | Yes | No | 89.3 | 1.89 |
| *Schaalia* | Yes | No | 97.3 | 1.73 |
| *Treponema* | Yes | No | 78.7 | 1.39 |
| *Peptostreptococcus* | Yes | No | 86.7 | 1.28 |
| *Pseudoleptotrichia* | Yes | No | 76.0 | 1.11 |
| *Rothia* | Yes | No | 90.7 | 1.06 |

Oral-associated genera were defined using body-site annotations from the expanded Human Oral Microbiome Database. The pathogen-associated set was prespecified based on the IDSA/ATS HAP/VAP framework and supplemented by respiratory microbiology and lower-airway microbiome literature. The two sets were not mutually exclusive; *Haemophilus* and *Serratia* were assigned to both. *Streptococcus* was retained in the HOMD-derived oral-associated set and excluded from the pathogen-associated set because genus-level V4 16S sequencing cannot reliably distinguish *Streptococcus pneumoniae* from closely related oral streptococci. Only oral-associated-only genera with a mean relative abundance of at least 1.0% across the 75 at-risk baseline BAL samples are shown; values below 0.01% are displayed as <0.01. SILVA v138.2 reports the *Escherichia* clade as *Escherichia-Shigella*.

**Table S4. Pairwise comparisons underlying the figure panels.**

| **Panel** | **Outcome** | **Contrast** | **Test** | **Adjusted *P*** |
| --- | --- | --- | --- | --- |
| Fig. 2B | Bray–Curtis dissimilarity | CST1 vs CST2 | PERMANOVA | 0.002 |
|  |  | CST1 vs CST3 |  | 0.004 |
|  |  | CST2 vs CST3 |  | 0.002 |
| Fig. 2D | Bacterial burden | CST1 vs CST2 | Dunn | <0.001 |
|  |  | CST1 vs CST3 |  | 0.003 |
|  |  | CST2 vs CST3 |  | 0.064 |
|  | Shannon diversity | CST1 vs CST2 | Dunn | <0.001 |
|  |  | CST1 vs CST3 |  | 0.001 |
|  |  | CST2 vs CST3 |  | 0.769 |
|  | Oral-associated proportion | CST1 vs CST2 | Dunn | <0.001 |
|  |  | CST1 vs CST3 |  | 0.285 |
|  |  | CST2 vs CST3 |  | <0.001 |
|  | Pathogen-associated proportion | CST1 vs CST2 | Dunn | 0.002 |
|  |  | CST1 vs CST3 |  | 0.871 |
|  |  | CST2 vs CST3 |  | 0.006 |
| Fig. 2E | Cumulative VAP incidence | CST1 vs CST2 | Gray | 0.025 |
|  |  | CST1 vs CST3 |  | 0.570 |
|  |  | CST2 vs CST3 |  | 0.164 |
| Fig. 3B | Bacterial burden between timepoints | T1 vs T2 | Wilcoxon signed-rank | 0.414 |
|  |  | T1 vs T3 |  | 0.414 |
|  |  | T1 vs T4 |  | 0.472 |
|  |  | T2 vs T3 |  | 0.249 |
|  |  | T2 vs T4 |  | 0.329 |
|  |  | T3 vs T4 |  | 0.572 |
| Fig. 3C | Declining-genus proportion over time | CST1 vs CST2 | Pairwise CST × timepoint interaction (LMM) | 0.004 |
|  |  | CST1 vs CST3 |  | 0.591 |
|  |  | CST2 vs CST3 |  | 0.012 |
| Fig. 4A | IL-1β | CST1 vs CST2 | Dunn | <0.001 |
|  |  | CST1 vs CST3 |  | 0.147 |
|  |  | CST2 vs CST3 |  | 0.018 |
|  | IL-6 | CST1 vs CST2 | Dunn | 0.027 |
|  |  | CST1 vs CST3 |  | 0.888 |
|  |  | CST2 vs CST3 |  | 0.041 |
|  | TNF-α | CST1 vs CST2 | Dunn | <0.001 |
|  |  | CST1 vs CST3 |  | 0.014 |
|  |  | CST2 vs CST3 |  | 0.046 |

Abbreviations: CST, community state type; LMM, linear mixed-effects model; T1–T4, sampling timepoints; VAP, ventilator-associated pneumonia.

Adjusted P values were obtained using the Benjamini–Hochberg method within each outcome. Dunn’s post hoc tests followed the corresponding Kruskal–Wallis tests. Pairwise PERMANOVA used the same Bray–Curtis distance matrix as the overall analysis, with 999 permutations. Pairwise Gray’s tests used the same competing-risk coding as the overall cumulative-incidence analysis. For Fig. 3B, paired Wilcoxon signed-rank tests were restricted to patients contributing samples at both timepoints; none of the six comparisons was significant after adjustment. For Fig. 3C, pairwise CST × timepoint interactions were estimated by refitting the linear mixed-effects model within each CST pair.

**Table S5. Relative and ddPCR-anchored estimated absolute abundance of the pathogen-associated genus set by community state type.**

**Panel A. Summary by community state type**

| **Community state type** | ***n*** | **Relative abundance, median (%)** | **Total bacterial burden, median (copies/μL)** | **Estimated absolute abundance, median (copies/μL)** |
| --- | --- | --- | --- | --- |
| CST1 | 33 | 6.6 | 4.5 × 10⁵ | 2.7 × 10⁴ |
| CST2 | 23 | 18.7 | 7.4 × 10² | 1.9 × 10² |
| CST3 | 19 | 9.1 | 2.1 × 10⁴ | 1.6 × 10³ |
| Kruskal–Wallis *P* |  | 0.001 | <0.001 | <0.001 |

**Panel B. Five leading pathogen-associated genera in CST2**

| **Genus** | **Mean relative abundance (%)** | **Median estimated absolute abundance (copies/μL)** | **Detected among CST2 non-VAP patients, *n*/15 (%)** | **Non-VAP mean relative abundance (%)** | **VAP mean relative abundance (%)** |
| --- | --- | --- | --- | --- | --- |
| *Escherichia-Shigella* | 6.4 | 2 | 13/15 (86.7) | 9.69 | 0.23 |
| *Haemophilus* | 5.4 | 18 | 14/15 (93.3) | 5.71 | 4.66 |
| *Staphylococcus* | 4.8 | 26 | 15/15 (100) | 5.37 | 3.58 |
| *Acinetobacter* | 4.1 | 35 | 15/15 (100) | 5.11 | 2.16 |
| *Klebsiella* | 3.9 | 24 | 14/15 (93.3) | 2.39 | 6.75 |

Abbreviations: BAL, bronchoalveolar lavage; CST, community state type; ddPCR, droplet digital PCR.

Estimated absolute abundance was calculated as relative abundance multiplied by the sample-matched ddPCR-derived total bacterial burden. Relative-abundance analyses included 75 baseline BAL samples and ddPCR-anchored analyses included 71. Panel B shows the five leading pathogen-associated genera in CST2. Genus membership is defined in Table S3.

**Table S6. Joint cause-specific Cox regression model including baseline community state type and bacterial-burden tertiles.**

| **Variable** | **Comparison** | **HR (95% CI)** | ***P*** |
| --- | --- | --- | --- |
| Community state type | CST1 vs CST2 | 4.29 (1.35–13.66) | 0.014 |
|  | CST3 vs CST2 | 2.96 (0.92–9.48) | 0.068 |
| Bacterial burden tertile | Middle vs low | 0.66 (0.23–1.87) | 0.433 |
|  | High vs low | 0.97 (0.37–2.58) | 0.952 |
| Sex | Male vs female | 0.93 (0.45–1.95) | 0.856 |
| GCS score | Per 1-point increase | 0.95 (0.84–1.08) | 0.413 |

Abbreviations: CST, community state type; GCS, Glasgow Coma Scale; HR, hazard ratio; VAP, ventilator-associated pneumonia.

The analysis included 70 patients with available baseline ddPCR measurements and 36 timed VAP events. The model included CST, bacterial-burden tertile, sex, and GCS; CST2, the low bacterial-burden tertile, and female sex were the reference categories. Adding CST to the model containing bacterial-burden tertile, sex, and GCS improved model fit (likelihood-ratio test, *P* = 0.041).

**Table S7. Pathogens cultured at VAP onset according to baseline community state type.**

| **Cultured pathogen** | **Overall *n* = 40** | | **CST1 *n* = 21** | **CST2 *n*= 8** | **CST3 *n* = 11** |
| --- | --- | --- | --- | --- | --- |
| ***Pathogen species*** | |  |  |  |  |
| *Staphylococcus aureus* | | 14 (35.0%) | 6 (28.6%) | 2 (25.0%) | 6 (54.5%) |
| *Enterobacter cloacae* | | 5 (12.5%) | 2 (9.5%) | 2 (25.0%) | 1 (9.1%) |
| *Klebsiella oxytoca* | | 5 (12.5%) | 3 (14.3%) | 1 (12.5%) | 1 (9.1%) |
| *Enterobacter aerogenes* | | 3 (7.5%) | 1 (4.8%) | 1 (12.5%) | 1 (9.1%) |
| *Escherichia coli* | | 2 (5.0%) | 2 (9.5%) | 0 (0.0%) | 0 (0.0%) |
| *Haemophilus influenzae* | | 2 (5.0%) | 1 (4.8%) | 0 (0.0%) | 1 (9.1%) |
| *Klebsiella pneumoniae* | | 2 (5.0%) | 1 (4.8%) | 1 (12.5%) | 0 (0.0%) |
| *Enterococcus faecalis* | | 1 (2.5%) | 1 (4.8%) | 0 (0.0%) | 0 (0.0%) |
| *Haemophilus parahaemolyticus* | | 1 (2.5%) | 1 (4.8%) | 0 (0.0%) | 0 (0.0%) |
| *Pseudomonas aeruginosa* | | 1 (2.5%) | 0 (0.0%) | 1 (12.5%) | 0 (0.0%) |
| *Proteus mirabilis* | | 1 (2.5%) | 1 (4.8%) | 0 (0.0%) | 0 (0.0%) |
| *Raoultella ornithinolytica* | | 1 (2.5%) | 1 (4.8%) | 0 (0.0%) | 0 (0.0%) |
| *Streptococcus pneumoniae* | | 1 (2.5%) | 0 (0.0%) | 0 (0.0%) | 1 (9.1%) |
| Other/unspecified | | 1 (2.5%) | 1 (4.8%) | 0 (0.0%) | 0 (0.0%) |
| ***Association with baseline CST*** | |  |  |  |  |
| Gram category, Fisher exact test | | *P* = 0.170 | | | |
| Species identity, simulated Fisher exact test | | *P* = 0.975 | | | |

Abbreviations: CST, community state type; VAP, ventilator-associated pneumonia.

Values are n (%) of culture-positive VAP episodes within each column. Percentages use the column total as the denominator. Species are ordered by overall frequency.

**Table S8. Baseline 16S detection of the genus subsequently cultured at VAP onset.**

| **Organism cultured at VAP onset (genus)** | **Episodes, *n*** | **Detected at baseline, *n* (%)** | **Background prevalence among non-VAP patients (%)** | **Median baseline relative abundance when detected (%)** | **Median baseline estimated absolute abundance when detected (copies/μL)** |
| --- | --- | --- | --- | --- | --- |
| *Staphylococcus* | 14 | 14 (100) | 91 | 0.64 | 370 |
| *Klebsiella* | 10 | 7 (70) | 71 | 0.57 | 25 |
| *Enterobacter* | 5 | 0 (0) | 0 | — | — |
| *Haemophilus* | 3 | 3 (100) | 97 | 6.5 | 58,000 |
| *Escherichia-Shigella* | 2 | 2 (100) | 63 | 11.3 | 91,000 |
| *Enterococcus* | 1 | 0 (0) | 34 | — | — |
| *Proteus* | 1 | 1 (100) | 31 | 0.04 | 88 |
| *Pseudomonas* | 1 | 1 (100) | 49 | 0.95 | 4.6 |
| *Raoultella* | 1 | 1 (100) | 3 | 0.03 | 14 |
| *Streptococcus* | 1 | 1 (100) | 100 | 43.6 | 320 |
| **Total** | **39** | **30 (77)** | **—** | **—** | **—** |

| **Test** | **Observed** | **Expected under the null** | ***P* value** |
| --- | --- | --- | --- |
| Detections versus prevalence-matched null | 30 | 26.3 | 0.056 |
| Mean genus-specific carriage percentile rank | 0.63 | 0.50 | 0.006 |
| Baseline detection by community state type | — | — | 1.000 |

Abbreviations: BAL, bronchoalveolar lavage; CST, community state type; ddPCR, droplet digital PCR; VAP, ventilator-associated pneumonia.

Restricted to 39 culture-positive VAP episodes with species-level identification; one additional episode reported as “other” was excluded. Cultured species were mapped to genus level and matched to the baseline BAL 16S profile of the same patient. Detection was defined as any non-zero read count and compared with a prevalence-matched null based on genus-specific prevalence among the 35 non-VAP patients (20,000 simulations). Genus-specific baseline abundance was assessed using percentile ranks tested against 0.50. *Enterobacter* was present in the reference taxonomy but was not detected in any baseline sample; V4 sequencing has limited resolution within the *Enterobacteriaceae*, and reads from these organisms may have been assigned to other genera of the same family.

**Table S9. Association of the declining-genus signature with subsequent VAP**

**Panel A. Is commensal loss faster in patients who develop VAP?**

| **Outcome scale** | **Effect** | **Estimate** | ***P* value** |
| --- | --- | --- | --- |
| CLR declining-genus score | Timepoint × VAP | −0.62 | 0.66 |
| Declining-genus proportion (%) | Timepoint × VAP | −4.10 | 0.046 |
| Absolute abundance of the declining-genus set (ddPCR-anchored) | Timepoint | −0.71 | <0.001 |
| Absolute abundance of the declining-genus set (ddPCR-anchored) | Timepoint × VAP | −0.48 | 0.27 |

**Panel B. Does the baseline commensal score predict VAP?**

| **Model** | **HR (95% CI)** | ***P* value** |
| --- | --- | --- |
| Baseline declining-genus score alone | 1.13 (0.78-1.61) | 0.52 |
| Baseline declining-genus score with community state type | 0.63 (0.36-1.08) | 0.09 |

Abbreviations: CLR, centered log-ratio; CST, community state type; ddPCR, droplet digital PCR; GCS, Glasgow Coma Scale; HR, hazard ratio; VAP, ventilator-associated pneumonia.

Panel A reports fixed effects from linear mixed-effects models with a patient-level random intercept, adjusted for sex and GCS. Estimated absolute abundance was calculated by multiplying the relative abundance of the declining-genus set by the sample-matched ddPCR-derived total bacterial burden. Panel B reports cause-specific Cox models adjusted for sex and GCS; hazard ratios are per 1-SD increase in the baseline CLR declining-genus score.

**Table S10. Genus-level baseline CST × timepoint interactions among dual-confirmed declining genera**

| **Genus** | **Estimated timepoint coefficient (β × 10³)** | | | **χ²** | ***P* value** | **FDR** |
| --- | --- | --- | --- | --- | --- | --- |
|  | **CST1** | **CST2** | **CST3** |  |  |  |
| *Rothia* | -3.7 | -1.8 | -6.0 | 9.40 | 0.009 | 0.020 |
| *Pseudoleptotrichia* | -3.8 | -0.7 | -6.6 | 8.95 | 0.011 | 0.020 |
| *Schaalia* | -6.7 | -0.4 | -4.2 | 11.40 | 0.003 | 0.020 |
| *Granulicatella* | -2.6 | -1.4 | -5.6 | 11.30 | 0.004 | 0.020 |
| *Veillonella* | -36.3 | -12.5 | -12.5 | 9.29 | 0.010 | 0.020 |
| *Gemella* | -5.3 | 0.1 | -11.3 | 8.91 | 0.012 | 0.020 |
| *Lachnoanaerobaculum* | -0.9 | 0.0 | -1.1 | 9.31 | 0.010 | 0.020 |
| *Stomatobaculum* | -1.2 | -0.1 | -1.7 | 5.12 | 0.077 | 0.116 |
| *Streptococcus* | -30.3 | -19.1 | -41.0 | 3.57 | 0.168 | 0.224 |
| *Bergeyella* | -1.1 | -0.1 | -1.5 | 3.15 | 0.207 | 0.248 |
| *Leptotrichia* | -1.7 | -0.6 | -1.5 | 2.57 | 0.276 | 0.301 |
| *Haemophilus* | -16.0 | -8.2 | -7.0 | 1.11 | 0.575 | 0.575 |

Abbreviations: CST, community state type; FDR, false discovery rate; LMM, linear mixed-effects model.

β values are CST-specific estimated timepoint coefficients from the full LMM and are multiplied by 10³ for readability. The outcome was log1p-transformed relative abundance. The CST × timepoint interaction was tested by likelihood-ratio comparison of the full model, y ~ timepoint × baseline CST + (1 | patient_ID), with the reduced model, y ~ timepoint + baseline CST + (1 | patient_ID). P values were adjusted across the 12 dual-confirmed declining genera using the Benjamini-Hochberg method. Negative β indicates decreasing relative abundance over time. In an aggregate sensitivity analysis of the declining-genus proportion, the baseline CST × timepoint interaction was significant on the percentage-point scale (*P*=0.003) but attenuated on the log scale (*P*=0.057); estimated proportional changes per timepoint were −42.7%, −24.9%, and −37.3% for CST1, CST2, and CST3, respectively.

**Table S11. Multivariable associations between baseline cytokines, bacterial burden, and community composition**

| **Cytokine** | **n** | **Predictor** | **β** | ***P* value** | **Unique variance (%)** | **Overall model R²** | **Overall model *P* value** |
| --- | --- | --- | --- | --- | --- | --- | --- |
| IL-1β | 68 | Bacterial burden | 0.240 | <0.001 | 32.5 | 0.47 | <0.001 |
|  |  | Declining-genus score | 0.019 | 0.141 | 2.2 |  |  |
| IL-6 | 68 | Bacterial burden | 0.038 | 0.318 | 1.4 | 0.14 | 0.045 |
|  |  | Declining-genus score | 0.024 | 0.034 | 6.6 |  |  |
| TNF-α | 68 | Bacterial burden | 0.135 | <0.001 | 27.6 | 0.43 | <0.001 |
|  |  | Declining-genus score | 0.014 | 0.08 | 3.3 |  |  |

Predictor refers to the microbiome-related explanatory variables of interest. Models were fitted separately for each cytokine as log₁₀(1 + cytokine concentration) ~ ln(1 + bacterial burden) + declining-genus score + sex + GCS. The declining-genus score was calculated from CLR-transformed abundances of the dual-confirmed declining genera after Bayesian-multiplicative zero replacement (zCompositions). β values are multivariable regression coefficients for the listed predictor. Bacterial burden and the declining-genus score were moderately correlated (Spearman ρ = 0.42; VIF = 1.4). Sex and GCS were included as adjustment covariates in all models but are not shown. Unique variance (%) was calculated from Type II sums of squares for each listed predictor. Overall model R² and overall model P value refer to the full cytokine-specific model and are shown once per cytokine.

**Table S12. Sensitivity analyses of CST assignment and VAP risk under alternative DMM strategies**

| **Analysis** | **State** | **Dominant primary CST** | **N** | **VAP, n (%)** | **Median bacterial burden** | **HR (95% CI)** | ***P* value** |
| --- | --- | --- | --- | --- | --- | --- | --- |
| Re-clustering at k = 2 | K2_2 | CST2, 22 (62.9%) | 36 | 13 (36.1) | 7,381 | Reference |  |
|  | K2_1 | CST1, 33 (84.6%) | 39 | 25 (64.1) | 442,776 | 2.26 (1.11-4.59) | 0.025 |
| Re-clustering at k = 4 | K4_3 | CST2, 19 (100%) | 19 | 5 (26.3) | 682 | Reference |  |
|  | K4_1 | CST1, 21 (100%) | 21 | 15 (71.4) | 986,234 | 5.14 (1.76-15.00) | 0.003 |
|  | K4_2 | CST3, 16 (76.2%) | 21 | 11 (52.4) | 16,861 | 2.98 (1.00-8.88) | 0.050 |
|  | K4_4 | CST1, 9 (69.2%) | 14 | 7 (50.0) | 442,776 | 2.35 (0.72-7.62) | 0.156 |
| Full 132-derived DMM (re-filtered features; seed 131) | State1 | CST2, 21 (87.5%) | 25 | 9 (36) | 805 | Reference |  |
|  | State2 | CST3, 15 (100%) | 15 | 9 (60.0) | 19,019 | 2.40 (0.95-6.08) | 0.065 |
|  | State3 | CST1, 32 (91.4%) | 35 | 20 (57.1) | 442,776 | 2.24 (1.00-5.00) | 0.049 |

Abbreviations: CST, community state type; DMM, Dirichlet multinomial mixture; VAP, ventilator-associated pneumonia; HR, hazard ratio; CI, confidence interval.

For k = 2 and k = 4 analyses, DMMs were refitted in the primary at-risk baseline cohort using the same genus-level feature set as the primary k = 3 model. Dominant primary CST indicates the most frequent primary k = 3 CST assignment within each alternative state.

For the full-cohort sensitivity analysis, DMM was refitted in all 132 baseline samples after re-applying the same genus filtering criteria. Because most random starts produced sparse states, the table reports a non-sparse solution from a 30-seed scan (seed 131); this analysis should be interpreted as exploratory rather than as independent confirmation of the primary CST solution.

Median bacterial burden is shown as ddPCR-derived 16S rRNA gene copies per microliter.

**Table S13. ASVs identified as contaminants by decontam (combined frequency–prevalence method, threshold = 0.1). A total of 46 ASVs were flagged; all entries are shown, ordered by total read count.**

| **#** | **Genus** | **Family** | **Total reads** | **decontam *P*** |
| --- | --- | --- | --- | --- |
| 1 | *Klebsiella* | Enterobacteriaceae | 73,793 | 0.0038 |
| 2 | *Streptococcus* | Streptococcaceae | 40,965 | 0 |
| 3 | *Prevotella* | Prevotellaceae | 36,693 | 0.0944 |
| 4 | *Lawsonella* | Corynebacteriaceae | 4,157 | 0.0058 |
| 5 | *Caulobacter* | Caulobacteraceae | 3,764 | 0 |
| 6 | *Anoxybacillus* | Bacillaceae | 3,707 | 0 |
| 7 | *Brochothrix* | Listeriaceae | 2,943 | 0.0497 |
| 8 | *Knoellia* | Intrasporangiaceae | 2,047 | 0.0015 |
| 9 | *Prevotella* | Prevotellaceae | 1,753 | 0.0915 |
| 10 | *Sphingobium* | Sphingomonadaceae | 1,666 | 0.003 |
| 11 | *Gemella* | Gemellaceae | 1,347 | 0.0231 |
| 12 | *Corynebacterium* | Corynebacteriaceae | 794 | 0.0883 |
| 13 | *Acinetobacter* | Moraxellaceae | 763 | 0.0135 |
| 14 | *Rubellimicrobium* | Paracoccaceae | 759 | 0.0182 |
| 15 | *Paracoccus* | Paracoccaceae | 710 | 0.0752 |
| 16 | *Tychonema CCAP 1459-11B* | Phormidiaceae | 652 | 0.0019 |
| 17 | *Empedobacter* | Weeksellaceae | 543 | 0.0565 |
| 18 | *Acinetobacter* | Moraxellaceae | 523 | 0.0327 |
| 19 | Unclassified | NA | 513 | 4e-4 |
| 20 | *Pseudomonas* | Pseudomonadaceae | 434 | 0.0822 |
| 21 | *Aerococcus* | Aerococcaceae | 416 | 0.0704 |
| 22 | *Peptostreptococcus* | Peptostreptococcaceae | 354 | 0.0643 |
| 23 | Unclassified | p-2534-18B5 gut group | 322 | 0.0951 |
| 24 | Unclassified | Neisseriaceae | 305 | 0.022 |
| 25 | *Veillonella* | Veillonellaceae | 244 | 0.0651 |
| 26 | *Treponema* | Spirochaetaceae | 211 | 0.0831 |
| 27 | *Sphingobium* | Sphingomonadaceae | 203 | 0.0897 |
| 28 | *Micrococcus* | Micrococcaceae | 198 | 0.0376 |
| 29 | *Niallia* | Bacillaceae | 173 | 0.0294 |
| 30 | *Lactobacillus* | Lactobacillaceae | 149 | 0.0679 |
| 31 | *Aurantisolimonas* | Chitinophagaceae | 127 | 0.061 |
| 32 | *Blastococcus* | Geodermatophilaceae | 124 | 0.0362 |
| 33 | *Massilia* | Oxalobacteraceae | 123 | 0.0321 |
| 34 | *Noviherbaspirillum* | Oxalobacteraceae | 110 | 0.0225 |
| 35 | *OLB17* | Blastocatellaceae | 109 | 0.0731 |
| 36 | *Chryseobacterium* | Weeksellaceae | 103 | 0.0029 |
| 37 | *Tannerella* | Tannerellaceae | 100 | 0.0378 |
| 38 | *Truepera* | Trueperaceae | 89 | 0.0122 |
| 39 | *Rubellimicrobium* | Paracoccaceae | 88 | 0.0497 |
| 40 | *Pseudomonas* | Pseudomonadaceae | 70 | 0.0362 |
| 41 | *Treponema* | Spirochaetaceae | 63 | 0.091 |
| 42 | *Lachnoanaerobaculum* | Lachnospiraceae | 52 | 0.047 |
| 43 | *Shuttleworthia* | Lachnospiraceae | 29 | 0.0802 |
| 44 | Unclassified | Anaerovoracaceae | 27 | 0.0534 |
| 45 | Unclassified | F082 | 22 | 0.0347 |
| 46 | *Leptotrichia* | Leptotrichiaceae | 14 | 0.031 |

**Table S14. Sequencing and bioinformatic pipeline summary.**

| **Step** | **Value** |
| --- | --- |
| DADA2 output (all samples incl. controls) | 1044 |
| Experimental samples | 874 |
| Non-experimental controls | 170 |
| Total ASVs (pre-decontam) | 13733 |
| Contaminant ASVs removed (decontam) | 46 |
| Organelle ASVs removed | 282 |
| ASVs removed after retained-sample filtering (zero reads) | 562 |
| ASVs retained | 12843 |
| Samples excluded (< 1,300 reads) | 35 |
| Experimental samples retained | 839 |
| BAL | 287 |
| Tracheal | 289 |
| Nasal | 263 |
| Genera after aggregation | 1202 |
| Genera (named) | 1007 |

**Supplemental Figure**

**Fig. S1. No baseline genus distinguishes future VAP from non-VAP.** Genus-level DESeq2 volcano plots in baseline BAL (at-risk cohort, n = 75): Model A (unadjusted) and Model B (adjusted for gender and GCS). The dashed line marks FDR = 0.05; orange points have unadjusted *P* < 0.05 but did not survive FDR correction. No genus reached FDR significance in either model.
